# Impact of doping on the mechanical properties of conjugated polymers

**DOI:** 10.1039/d3cs00833a

**Published:** 2024-01-24

**Authors:** Sri Harish Kumar Paleti, Youngseok Kim, Joost Kimpel, Mariavittoria Craighero, Shuichi Haraguchi, Christian Müller

**Affiliations:** a Department of Chemistry and Chemical Engineering, Chalmers University of Technology 41296 Göteborg Sweden; b Wallenberg Wood Science Center, Chalmers University of Technology 41296 Göteborg Sweden christian.muller@chalmers.se

## Abstract

Conjugated polymers exhibit a unique portfolio of electrical and electrochemical behavior, which – paired with the mechanical properties that are typical for macromolecules – make them intriguing candidates for a wide range of application areas from wearable electronics to bioelectronics. However, the degree of oxidation or reduction of the polymer can strongly impact the mechanical response and thus must be considered when designing flexible or stretchable devices. This tutorial review first explores how the chain architecture, processing as well as the resulting nano- and microstructure impact the rheological and mechanical properties. In addition, different methods for the mechanical characterization of thin films and bulk materials such as fibers are summarized. Then, the review discusses how chemical and electrochemical doping alter the mechanical properties in terms of stiffness and ductility. Finally, the mechanical response of (doped) conjugated polymers is discussed in the context of (1) organic photovoltaics, representing thin-film devices with a relatively low charge-carrier density, (2) organic thermoelectrics, where chemical doping is used to realize thin films or bulk materials with a high doping level, and (3) organic electrochemical transistors, where electrochemical doping allows high charge-carrier densities to be reached, albeit accompanied by significant swelling. In the future, chemical and electrochemical doping may not only allow modulation and optimization of the electrical and electrochemical behavior of conjugated polymers, but also facilitate the design of materials with a tunable mechanical response.

## Abstract 2

Chemical and electrochemical doping can strongly alter the electrical but also mechanical properties of conjugated polymers, which may enable new types of tunable materials and devices of interest for wearable electronics and bioelectronics.

## Graphical abstract

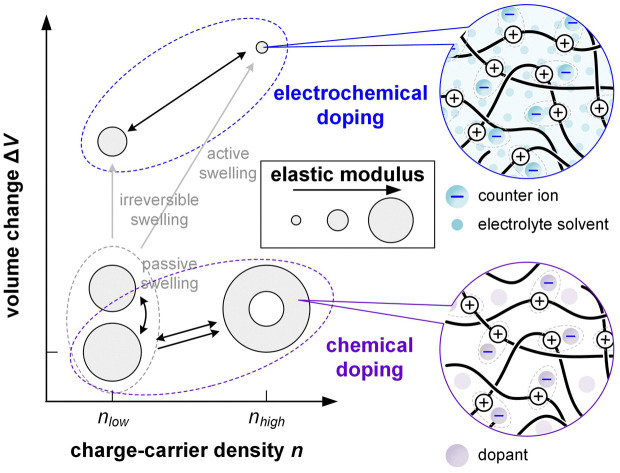

**Key learning points**

– Conjugated polymer-based materials cover the full spectrum of mechanical behavior from stretchable polymers and elastic blends to stiff composites.

– The mechanical properties of polymers depend on the processing history and the resulting nano- and microstructure, and should only be compared if measured with the same bulk or thin-film measurement technique.

– Chemical and electrochemical doping can strongly alter the rheological and mechanical properties of initially soft conjugated polymers, while materials with a high elastic modulus are less affected.

– The electrical and mechanical properties of conjugated polymers such as the electrical conductivity and elastic modulus tend to correlate but can be partially decoupled through the use of multi-component systems and the addition of suitable dopants.

– In the case of devices that operate at low charge-carrier densities, the mechanical properties of the undoped semiconductor should be considered, while the properties typical for doped polymers govern the behavior of highly charged devices.

## 1. Introduction

Conjugated polymers are an intriguing class of materials that are widely used for the design of flexible electronic devices. One motivation is the notion that their polymeric nature naturally imparts advantageous mechanical properties such as flexibility, ductility and/or stretchability, which sets them apart from, *e.g.*, carbon allotropes or inorganic semiconductors. However, the conjugated backbone and prevalence of aromatic units tend to result in relatively rigid and low molecular-weight materials, which do not exhibit the desired portfolio of mechanical properties. Hence, a number of strategies from side-chain engineering to copolymerization and blending have been established that allow improvement of the ductility and flexibility of conjugated polymers (Section 2).

The mechanical properties of conjugated polymers change when charge carriers are introduced because of a change in the rigidity of the backbone upon oxidation or reduction, but also because of the counterions that are often introduced to balance the charge on the polymer. The function of many types of thin-film as well as bulk electronic devices involves the modulation of the charge-carrier density, which can also alter the mechanical properties during operation. Depending on the dimensions of the polymer film, tape or fiber that is used to construct various devices, different types of measurements can be used to elucidate the thin-film or bulk mechanical response (Section 3).

**Fig. 1:**
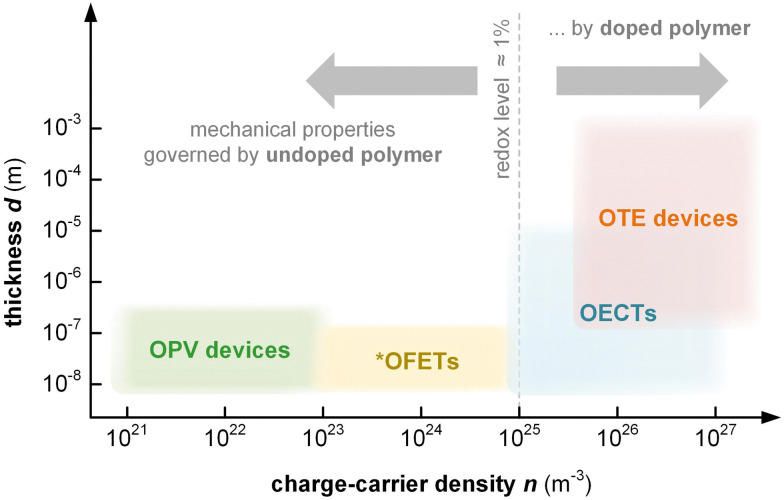
Approximate range of charge-carrier density *n* and active-layer thickness *d* of organic photovoltaic (OPV) devices, organic field-effect transistors (OFETs), organic electrochemical transistors (OECTs) and organic thermoelectric (OTE) devices. *^ ^Average *n* for entire volume of semiconductor film; charges accumulate at the interface with the gate dielectric.

Thin-film devices such as organic light emitting diodes (OLEDs), organic solar cells and organic field-effect transistors (OFETs) employ conjugated polymers in their semiconducting state and then modulate their charge-carrier density through the application of an electric potential or through the exposure to an external stimulus such as light. In addition, modest chemical doping of thin-film devices can be used to improve charge transport through trap filling and to reduce contact resistance effects.^1,2^ The charge-carrier density in OLEDs and organic photovoltaic (OPV) devices typically reaches values of 10^21^ to 10^23^ m^−3^ (Fig. 1),^3^ which implies that only few sites are charged (total number of sites about 10^27^ m^−3^). In the case of OFETs higher values are reached but charges accumulate in a nanometer thin region at the interface with the gate dielectric,^1^ meaning that most of the material remains uncharged. Hence, to understand the mechanical response of conjugated polymer films in thin-film devices, their semiconducting (or weakly doped) state should be considered (Section 4).

Instead, bulk devices such as organic thermoelectric (OTE) generators employ conjugated polymers in a strongly oxidized or reduced state with charge-carrier densities in the range of 10^26^ to 10^27^ m^−3^ (Fig. 1), often brought about *via* chemical doping (Section 5).^2^ Other types of thin-film devices such as organic electrochemical transistors (OECTs)^4^ or bulk devices such as polymer actuators^5^ modulate the electrical properties of the polymer *via* electrochemical doping, with the conducting state often exhibiting a very high charge-carrier density of up to 10^27^ m^−3^ (Fig. 1), which is only limited by the high degree of swelling that the material experiences (*cf.* Section 6). For such highly charged devices the mechanical properties of the doped (and swollen) polymer are decisive.

This review will first explore the configuration and conformation of conjugated polymers from a synthesis and structure–processing–property perspective (Section 2). Then, various techniques are discussed that can be used to study the mechanical properties of thin-films or bulk materials (Section 3), which are composed of either the neat semiconductor or a multi-component system of the conjugated polymer together with an acceptor or dopant molecule, another conjugated or an insulating polymer, or a reinforcing agent such as a carbon allotrope or a nanocellulose particle (Section 4). Emphasis is put on the impact of chemical and electrochemical doping on the mechanical properties of conjugated polymers (Sections 5 and 6). Finally, the mechanical properties are discussed in the context of different applications, with focus on organic solar cells as an example of a device where the undoped semiconductor is more relevant, as well as OTE devices and OECTs, which rely on highly chemically or electrochemically doped materials, respectively (Section 7).

## 2. Synthesis, conformation and nanostructure of conjugated polymers

**Fig. 2:**
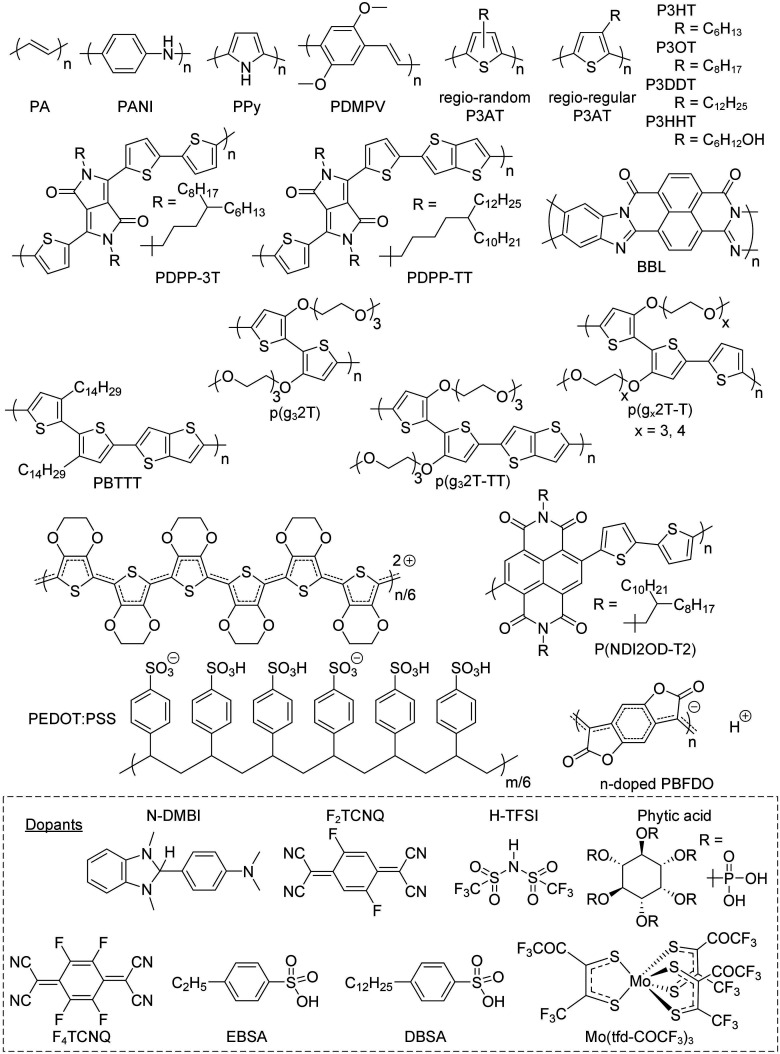
Chemical structures of the conjugated polymers and dopants discussed in this review.

Conjugated polymers feature a conjugated backbone that comprises a polyacetylene skeleton (PA; Fig. 2). Since PA is characterized by poor environmental stability, stabilization of double bonds is required, usually achieved through the incorporation of aromatic ring motifs that result in a rigid polymer chain. Hence, unsubstituted conjugated polymers that only comprise a neutral conjugated backbone tend to be intractable. The two main approaches to impart processability from solution and/or melt increase the conformational entropy of the overall system and involve (1) the preparation of the polymer in its oxidized form, paired with solubilizing counterions, and (2) the decoration of the backbone with flexible side chains. The chain configuration in terms of repeat unit(s) and side chain(s) impacts the choice of synthesis and processing routes, and *vice versa* (Section 2.1.), as well as the chain conformation (Section 2.2), which in turn influences the nanostructure (Section 2.3) and thus the electrical and mechanical properties of the resulting conjugated polymer.

### 2.1. Synthesis of conjugated polymers

**Fig. 3:**
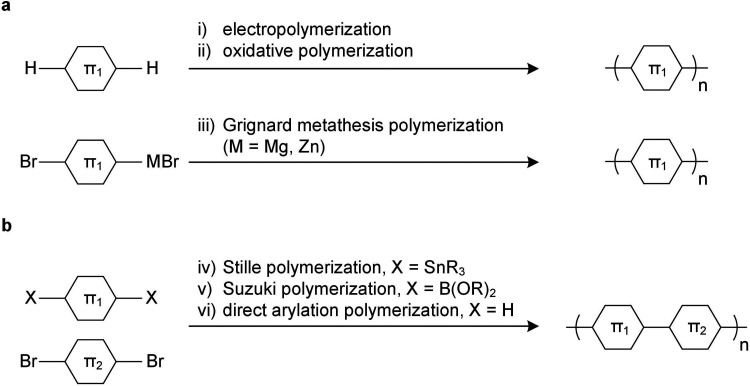
Polymerization methods using (a) a single monomer and (b) two monomers.

Since unsubstituted conjugated polymers are difficult to process, an early preparation route involved electropolymerization (Fig. 3a), which combines synthesis and film formation (processing) in one single step. Monomers in a reaction medium undergo electron transfer with an electrode, forming a radical cationic monomer that reacts with the next monomer, and so forth, resulting in a conjugated polymer film on the electrode. The obtained polymer is insoluble, meaning that electropolymerized materials tend to be poorly characterized.

Alternatively, unsubstituted conjugated polymers can be prepared by chemical oxidative polymerization and are processable in their oxidized form provided suitable counterions are chosen that impart solubility. The most prominent example is oxidative polymerization of poly(3,4-ethylenedioxythiophene) (PEDOT) in the presence of polystyrene sulfonate (PSS).^6^ The resulting polymer:polyanion PEDOT:PSS complex can be processed as a dispersion from water, yielding p-type conducting films or fibers with an electrical conductivity of up to 3500 S cm^−1^ and a Young's modulus *E* = 22 GPa (Section 5).^7^ Recently, a similar approach has been reported for poly(benzodifurandione) (PBFDO), which involved oxidative polymerization and *in situ* reductive n-doping, resulting in a polymer:proton complex that can be processed from dimethyl sulfoxide (DMSO),^8^ which can be wet-spun into n-type conducting fibers with an electrical conductivity of up to 1600 S cm^−1^ and a Young's modulus *E* = 19.5 GPa.^9^

The introduction of side chains greatly enhances the solubility of growing polymer chains, which has led to the development of a plethora of polymerization techniques for conjugated polymers.^10,11^ Polymers with a simple repeat unit such as poly(3-alkylthiophene)s (P3ATs) can be prepared by chemical oxidative polymerization, which yields a regio-random and thus disordered polymer with a low elastic modulus (Section 4). Instead, Kumada (McCullough method) or Negishi coupling (Rieke method) can be used, involving monosubstituted organomagnesium halide (Grignard) or organozinc halide reagents (Fig. 3a), which permit the synthesis of regio-regular P3ATs that feature an elastic modulus of several 100 MPa at room temperature (Section 4). Kumada coupling can result in high-molecular weight polymers, *e.g.* P3HT with a number-average molecular weight *M*_n_ >300 kg mol^−1^ has been reported,^12^ which is significantly larger than the entanglement molecular weight (see Section 2.3).^13^

Polymers with more complex repeat units can be realized by combining two monomers, usually done *via* polycondensation methods using precious metal catalysts.^10,11^ A dibrominated aromatic unit undergoes polymerization with difunctionalized aromatic comonomers through Stille coupling (diorganotin), Suzuki coupling (diorganoboronate) or Kumada coupling (diorganomagnesium) (Fig. 3b). Some monomers feature sufficiently active aromatic C–H bonds, which enable direct arylation polymerization through reaction with a dibrominated aromatic monomer (Fig. 3b).^14^

The solubility of a polymer generally decreases with the degree of polymerization, and many conjugated polymers already become insoluble once even modest molecular weights are reached, which complicates synthesis, workup, characterization and, finally, processing. Molecular weight control in polycondensation reactions is – according to Carother's equation – linked to the stoichiometric ratio of the two monomers involved in the coupling reaction. Any stoichiometric imbalance means that at high monomer conversion the majority of chain ends are populated by the reactive groups associated with the excess monomer, which hinders any further reaction, leading to shorter chains. Hence, a stoichiometric imbalance can be deliberately selected to limit the molecular weight of the polymer, ensuring that the prepared material remains soluble. Conversely, high molecular weights are difficult to achieve with polycondensation reactions because (1) a 1 : 1 stoichiometric ratio is difficult to ensure and (2) the solubility of high-molecular-weight conjugated polymers is limited, which explains why only few conjugated polymers feature a high level of ductility (Section 4.2).

**Fig. 4:**
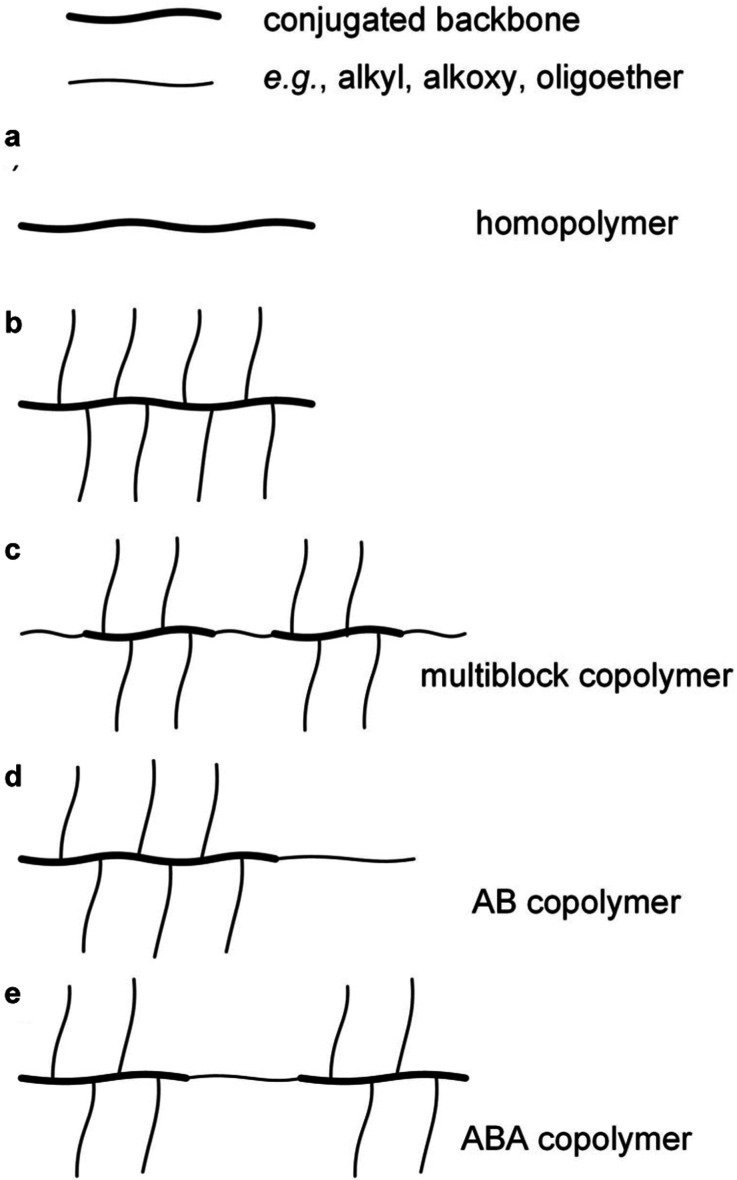
Conjugated polymer-based architectures of (a) a unsubstituted homopolymer, (b) a homopolymer with a conjugated backbone and flexible side chains, (c) a multiblock copolymer comprising conjugated segments separated by flexible spacers, and (d) AB and (e) ABA type block copolymers comprising conjugated and flexible (insulating) blocks.

Conjugated repeat units can be incorporated into the full spectrum of copolymer architectures, either with other conjugated repeat units and/or with non-conjugated parts. Random copolymerization involving two or more conjugated comonomers, again usually through polycondensation reactions, is widely used to combine the optical and electrical properties of the respective homopolymers. A variety of regular copolymer architectures have been described such as random or alternating multiblock copolymers comprising conjugated segments separated by flexible, non-conjugated spacers, which is a widely used approach for adjusting the mechanical properties of conjugated polymers (Fig. 4).^15,16^ Moreover, conjugated and non-conjugated polymers can be combined in different ways to create copolymers with different types of mechanical response. For instance, P3HT blocks have been combined with a polyethylene block resulting in AB type block copolymers that display greatly enhanced ductility^17^ but retain their electronic properties even if the conjugated block only comprises as little as 10 wt% of the copolymer.^18^ Another intriguing example are ABA type copolymers comprising two P3HT blocks connected *via* a flexible polymethacrylate (PMA) block, resulting in a material with a nanostructure that is typical for thermoplastic elastomers.^19^

### 2.2. Chain conformation of conjugated polymers

The stiffness of a polymer chain is described by its persistence length *l*_p_, which corresponds to half the length of a hypothetical chain segment – the so-called Kuhn length – that can be considered as freely joined, *i.e.* the orientation of adjacent segments is uncorrelated. The conformational space of a polymer chain that is much longer than *l*_p_ can be described by a random coil, while very short chains behave akin to a rigid rod. Common conjugated polymer building blocks such as five and six-membered aromatic rings (*e.g.* thiophene, benzene) as well as fused rings (*e.g.* thienothiophene) are characterized by small deflection angles of 0 to 15° and high rotational energy barriers. Hence, conjugated polymers feature semiflexible to rigid rod-like backbones with a high *l*_p_, resulting in a low conformational entropy in the liquid and molten state and a tendency to display liquid-crystalline order. A thorough discussion of the rigidity of conjugated polymers can be found elsewhere.^20^

A rigid backbone implies that the entropy change upon dissolution or melting is low leading to high dissolution and melting temperatures, which can limit polymerization as well as processing. Some unsubstituted polymers can be processed when oxidized with suitable dopants. For instance, polyaniline (PANI) becomes soluble in organic solvents and can be melt-processed upon blending with a variety of commodity polymers when protonated with, *e.g.*, dodecylbenzenesulfonic acid (DBSA),^21^ where the alkyl chain increases the overall conformational entropy of the polymer:counterion complex.

A more common approach to facilitate polymerization (*cf.* Section 2.1) and impart processability is the decoration of the conjugated backbone with flexible alkyl or oligoether side chains (Fig. 4), again leading to a higher overall conformational entropy even though in some cases long side chains can actual increase *l*_p_.^22,23^ As a result, neutral polymers with an appreciable degree of polymerization (molecular weight) remain soluble in the reaction medium as well as the solvent(s) chosen for workup, characterization and, finally, processing.

The optimal side-chain length represents a compromise between solubility, which benefits from longer side chains, and maximizing the fraction of the (opto)electronically active conjugated part, which implies that side chains should be short. In case of P3ATs, for instance, hexyl side chains have an optimal length, resulting in a soluble material with good charge transport characteristics.^24^ However, if mechanical properties are also considered slightly longer heptyl side chains may be preferable because the glass transition temperature *T*_g_ is lowered to below room temperature, which results in a significant reduction in elastic modulus (Section 4).^25^

Most conjugated polymers don alkyl, alkoxy, thioalkyl or oligoether side chains that are either linear or branched. Side chains can be functionalized with, *e.g.*, sulfonic or carboxylic acid groups, amines, urethane and ester groups, which can introduce intermolecular interactions such as ionic or hydrogen bonds. Moreover, crosslinkable moieties can be added, which facilitate the formation of covalent network points (see Section 4.2).

### 2.3. Nanostructure of conjugated polymers

Conjugated polymers share many features with commodity polymers but also display distinctive behavior that arises due to a strong tendency for π-stacking and the ubiquitous presence of side chains. Ordered domains typically feature π-stacking of polymer backbones with side chains oriented orthogonal to the π-stacking direction, separating backbones with the lamellar distance determined by the side-chain length. Thin films tend to be textured with π-stacking either preferentially in-plane (edge-on orientation) or out-of-plane (face-on orientation) with respect to the substrate. Further, uniaxial alignment can be achieved both in thin films and bulk materials (*cf.* Section 4).

The nanostructure of relatively flexible polymers such as regio-regular P3HT, which has a persistence length of *l*_p_ ≈ 3 nm at room temperature in dichlorobenzene,^26^ in many ways resembles that of polyethylene (modelling of the chain conformation yielded a similar value of *l*_p_ ≈ 4 nm^27^). P3HT chains can fold but when crystallized from the melt tend to organize in a fringed-micelle type nanostructure with crystalline domains that are separated by rigid amorphous as well as amorphous regions.^28^ Crystalline domains are connected *via* tie chains, provided the molecular weight is sufficiently high, *i.e.* number-average molecular weight *M*_n_ ≥ 25 kg mol^−1^ in case of regio-regular P3HT, leading to a ductile material with a tensile elastic modulus *E* of about 0.2 MPa and charge-carrier mobility *μ* < 0.1 cm^2^ V^−1^ s^−1^ (Section 4).^13,29^ The molecular-weight distribution strongly impacts the probability of tie-chain formation and concomitantly charge transport in thin films.^30^ The regio-regularity dictates to which extent the polymer can order. The strong tendency to π-stack can result in the growth of highly elongated, fibril-like crystallites, which can form individual “whiskers” or “nanofibrils” when solidified from dilute solution.^31^

More rigid conjugated polymers such as PBTTT, which has a persistence length of *l*_p_ ≈ 4–5 nm in chlorobenzene at room temperature,^32^ display a liquid–crystalline phase above the melting temperature *T*_m_, which facilitates the development of extended ordered domains, resulting in a higher *μ* > 0.3 cm^2^ V^−1^ s^−1^ and *E* ≈ 1.8 GPa (buckling method) compared with P3HT.^29^ Many conjugated polymers have a large persistence length that does not favor chain folding. Some rigid conjugated polymers do not feature any long-range order when studied with X-ray diffraction and should instead be thought of as comprising somewhat ordered regions with varying size and degrees of *para*-crystallinity, that are embedded in a disordered matrix.^33^

One class of rigid materials are diketopyrrolopyrrole (DPP) based copolymers such as PDPP-3T (see Fig. 2 for chemical structure), which has a persistence length of *l*_p_ ≈ 16 nm in *o*-dichlorobenzene at room temperature^23^ and thus is only able to form a fringed-micelle type nanostructure. PDPP-3T with an intermediate molecular weight of about *M*_n_ ≈ 90 kg mol^−1^ features a nanostructure comprising ordered regions that are connected by tie chains, resulting in a peak in *E* of 460 MPa (buckling method; 700 MPa if measured with force microscopy; *cf.* Section 3) and *μ* ≈ 4 cm^2^ V^−1^ s^−1^.^34^ Instead, lower molecular-weight material features chain-extended crystals but no connectivity, while entanglements of higher molecular weight PDPP-3T hinder the formation of ordered regions, both resulting in a decrease in *E* and *μ*. Attempts to improve the poor ductility of DPP-based copolymers by introducing, *e.g.*, hydrogen-bonding moieties have been met with limited success. The introduction of 10% amide- or urea-containing side chains does not strongly influence *E* but can alter the strain at break *ε*_break_, measured with the film-on-water method (see Section 3.2), which appeared to occur because of the concomitant change in the degree of order.^35^

## 3. Methods for measuring the rheological and mechanical properties

A range of techniques exist for characterizing the mechanical properties of bulk materials and thin films. Since results from different techniques can vary, a comparison of properties should only be done if measurements were carried out in a similar fashion, *i.e.* using the same sample preparation method and sample dimensions, as well as the same mode of deformation, temperature and frequency.

### 3.1. Bulk measurements

Mechanical measurements of commodity polymers are typically carried out using bulk measurement techniques that require relatively large amounts of material (at least 0.1–1 g per sample). Hence, classical techniques are, in many cases, of limited use for conjugated polymers since most materials are only available in small amounts.

#### 3.1.1. Tensile testing

**Fig. 5:**
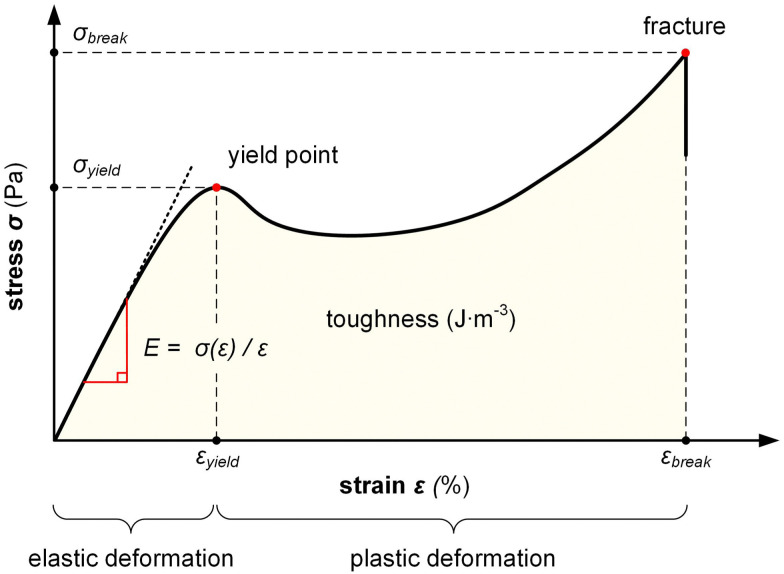
Stress–strain curve recorded during a tensile deformation experiment of a ductile polymer allows to determine the Young's modulus *E*, yield strain and stress, *ε*_yield_ and *σ*_yield_, the strain and stress at break, *ε*_break_ and *σ*_break_, and the toughness (area under *σ*(*ε*) curve).

Tensile testing is the most widely used technique for investigating the mechanical properties of polymers and can be used to determine the stiffness, ductility, elasticity, stretchability and toughness of a material (Table 1; see text box for definition of mechanical properties). A sample such as a dog-bone shaped specimen, a tape or fiber is drawn in one direction, typically at a constant (static) strain rate (strain-controlled mode), which allows to record the stress *σ* as a function of strain *ε* = (*l* − *l*_0_)/*l*_0_ = Δ*l*/*l*_0_ until fracture (Fig. 5), where *l*_0_ and *l* are the initial length and the length of the stretched sample, respectively. The experiment is typically carried out with a tensile tester but can also be performed with a dynamic mechanical analyzer in tensile mode (see Section 3.1.2), which can be advantageous for conjugated polymers because small samples can be analyzed. Besides commercial instruments, it is possible to construct benchtop setups or even design an apparatus with LEGO bricks.^36^

**Table 1:** Summary of the most common techniques used to characterize the mechanical properties of conjugated polymers (samples with different thickness *d*, free-standing or supported), some of which can be used to carry out variable-temperature measurements (*e.g.* to determine the *T*_g_) and provide information about the elastic properties (*e.g.* tensile or shear elastic modulus *E*′ or *G*′) and/or plastic deformation (*e.g.* strain at break *ε*_break_ and crack onset strain *ε*_crack_) as well as swelling

	Support	*d*	*T*_g_	*E*′ or *G*′	*ε*_break_	*ε*_crack_	Swelling
Tensile testing	—	≫μm	(✓)	✓	✓	—	—
DMA	—	≫μm	✓	✓	(✓)	—	—
DMA, fiber mesh	✓	<μm	✓	—	—	—	—
DMA, elastic support	✓	<μm	✓	✓	(✓)	—	—
Shear rheometry	✓	≫μm	✓	✓	—	—	—
QCM-D	✓	<μm	—	(✓)	—	—	✓
Nanoindentation	✓	≈μm	(✓)	✓	—	—	✓
AFM	✓	<μm	—	✓	—	—	✓
Buckling	✓	≪μm	—	✓	—	(✓)	—
Film-on-water tensile testing	✓	≪μm	—	✓	✓	✓	—

The stress *σ* = *F*(*ε*)/*A*, *i.e.* the applied force per cross sectional area of the sample, can be expressed as engineering stress where the area is the initial area, *A* = *A*_0_, and true stress if the area corresponds to the actual area *A* = *A*(*ε*). Initially, the sample experiences elastic deformation and the material would return to its original shape if the stress was removed at this point. Stress increases linearly with strain *ε*, and the slope is referred to as the Young's modulus *E* = *σ*(*ε*)/*ε* (note that for static deformation the symbol *E* is written without an apostrophe; *cf.* Section 3.1.2). The tensile stiffness of the sample depends on *E* as well as the geometry of the sample according to:1
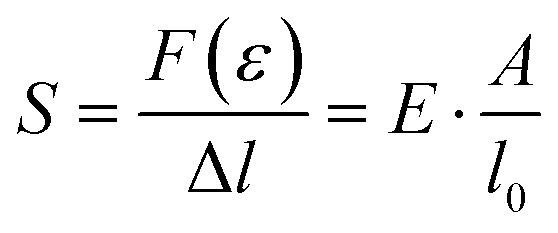


Tensile deformation beyond the yield stress *σ*_yield_ results in plastic (permanent) deformation, *i.e.* the material would not recover its original shape if the stress was removed. Hence, stretching can be used to produce polymer tapes and fibers with a high degree of permanent orientation, which has been exploited for aligning a wide range of conjugated polymers including PA, P3ATs, poly(*p*-phenylene vinylene)s (PPVs) as well as various blends comprising conjugated polymers and insulating polymers, including polyethylene and polyaramide.^37^

Ultimately, the sample breaks, yielding the strain and stress at break, *ε*_break_ and *σ*_break_. A material with a low *ε*_break_ is referred to as brittle, while ductile materials feature an *ε*_break_ ≫ 100%. The area under the stress–strain curve is the toughness, *i.e.* the energy per volume absorbed by a material during tensile deformation, meaning that an initially stiff and then ductile material has a high toughness.

**Definition of mechanical properties**

Stiffness (stiff): the ability of a material to resist deformation. Flexibility (flexible): inverse of stiffness. The ability of a material to deform. Elasticity (elastic): the ability of a material to reversibly deform under application of a mechanical stress, including the return to its original shape once the stress is removed. Ductility (ductile): the ability of a material to irreversibly deform under application of a mechanical stress without fracture. Yield and tensile strength (strong): the maximum stress that a material can experience without plastic deformation (yield) and fracture, respectively. Stretchability (stretchable): the ability of a material to reversibly *or* irreversibly deform through tensile drawing. Toughness (tough): the amount of energy per volume that a material can absorb during deformation prior to fracture.

#### 3.1.2. Dynamic mechanical analysis (DMA)

**Fig. 6:**
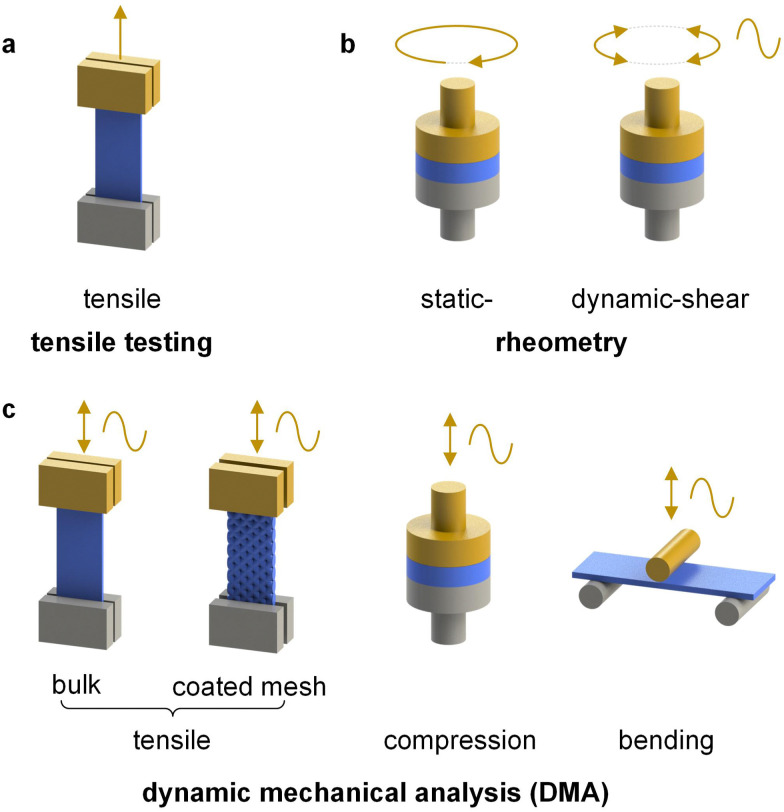
Selected modes of deformation available *via* (a) tensile testing, (b) rheometry and (c) dynamic mechanical analysis (DMA); the moving part is shown in yellow.

Dynamic mechanical analyzers can operate with different deformation modes such as tension, compression, bending and torsion (Fig. 6). It is possible to carry out static experiments by applying a gradually increasing strain or force (strain- or force-controlled mode) and thus achieve a continuous deformation such as a miniature tensile drawing experiment (see Section 3.1.1). Instead, dynamic experiments are done by applying a small oscillatory strain or force, which allows to study changes in modulus as a function of temperature and frequency (see also Section 3.1.3). Dynamic mechanical analysis (DMA) is well suited for investigating the mechanical properties of conjugated polymers because relatively small samples are required, *e.g.* several millimeter long tapes or fibers, which can be prepared with 10–50 mg of material.^38,39^ DMA can be carried out with even smaller amounts of material on the order of 5–10 mg by using so-called materials pockets.^40^

Alternatively, a thin film of the conjugated polymer can be deposited on a glass fiber mesh,^41^ an elastic substrate made of, *e.g.*, polydimethylsiloxane (PDMS)^42^ or a kirigami-cut polyimide film^43^ but only the latter two can provide absolute measurements of the elastic modulus. In case of fiber-mesh supported samples the cross-sectional area is ill-defined since the mesh and sample are entwined with each other. Hence, the glass fiber mesh method only allows to determine relative changes in storage and loss modulus, which is however widely used for the determination of the *T*_g_ of conjugated polymers and organic photovoltaic blends (Table 1).^41,44–46^

**Fig. 7:**
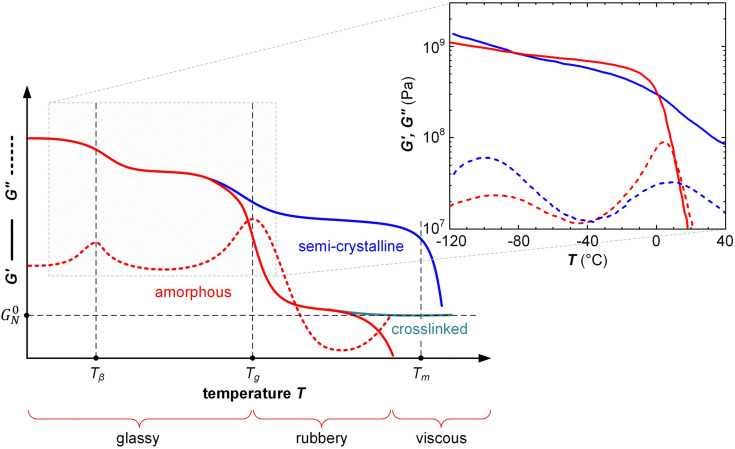
Typical DMA thermograms of the storage and loss moduli, *G*′ (solid) and *G*′′ (dashed), *vs.* temperature *T* of an amorphous polymer (red), semi-crystalline polymer (blue) and crosslinked, amorphous polymer (turquoise) with rubber plateau modulus *G*^0^_N_, featuring a β-relaxation temperature *T*_β_, glass transition temperature *T*_g_ (also referred to as α-relaxation temperature *T*_α_) and melting temperature *T*_m_; the inset shows *G*′ and *G*′′ of a regio-random (red; *M*_n_ = 42 kg mol^−1^, polydispersity index PDI = 2.4) and regio-regular P3HT (blue; *M*_n_ = 24 kg mol^−1^, PDI = 1.2, regio-regularity = 95%) measured with oscillatory shear rheometry at a frequency of 10 rad s^−1^; data reproduced from ref. 47.

Oscillatory deformation is carried out within the linear viscoelastic regime (typically, the strain amplitude is much less than 1%) so that the sample experiences no permanent (plastic) deformation at each temperature and frequency *f* = *ω*/2π where a measurement is carried out. The oscillating strain and stress are given by *ε*(*t*) = *ε*_0_ sin(*ωt*) and *σ*(*t*) = *σ*_0_ sin(*ωt* + *δ*), respectively, where *δ* is the phase lag. For a perfectly elastic material, *e.g.* a glassy or rubbery material, the phase lag *δ* = 0, *i.e.* the material responds instantaneously to a change in strain or stress. Conversely, any viscous component, which is most prominent when a material transitions from the glassy to the rubbery regime, and *vice versa*, leads to the dissipation of energy and a phase lag *δ* ≠ 0. The storage (elastic) and loss modulus are given by *G*′ = *σ*_0_/*ε*_0_ × cos *δ* and *G*′′ = *σ*_0_/*ε*_0_ × sin *δ*. Note that the symbols *G*′ and *E*′ are typically used for dynamic shear or tensile deformation, respectively (see Section 3.1.3). The loss tangent is defined as:

2 tan *δ* = *G*′′/*G*′

and can be determined by measuring the lag between stress and strain, allowing to compare the relative contribution of the elastic and viscous response with tan *δ* = 0 for a perfectly elastic material and tan *δ* ≫ 0 for a viscous material. Further, the *T*_g_ as well as sub-glass transition processes, which are prominent in many conjugated polymers because of their long solubilizing side chains,^38,46^ can be easily detected by identifying the temperature(s) at which tan *δ* or *G*′′ peak during a variable-temperature DMA measurement (Fig. 7).

#### 3.1.3. Rheometry

There are two types of instruments, extensional and rotational rheometers. Extensional rheometers, which apply a linear deformation, provide information about the viscoelastic response of polymer melts, such as strain hardening, but are not used for the characterization of conjugated polymers, since those are typically processed from solution. Rotational rheometers allow to apply an oscillatory or static rotational deformation to a disk-shaped sample. The torque needed to achieve a certain degree of angular displacement is recorded, which allows to calculate the shear stress *τ* and strain *γ*. Samples are relatively small, only 5–20 mg of material is required, and hence rotational rheometry is well suited for studying conjugated polymers. Rotational rheometry can provide information about the viscoelastic response of soft solids, polymer melts and liquids and is thus widely used for investigating the viscous properties of the solutions that are used to process conjugated polymers.

Entangled polymer melts and solutions feature a rubber plateau modulus *G*^0^_N_ and a rheometer can hence be used to study the impact of entanglements on the mechanical properties of conjugated polymer melts and solutions (see Section 4). For example, static shear rheometry has been used to record the viscosity of P3HT solutions as a function of solvent type and polymer molecular weight, which allowed to investigate the impact of chain entanglement on pre-aggregation.^48^ Oscillatory shear rheometry can be used to study gel formation in conjugated polymer solutions by identifying the crossover point where the shear elastic (storage) and loss modulus are of equal magnitude, *G*′ = *G*′′; for a viscous liquid *G*′ < *G*′′ while for a gel the elastic response dominates, *i.e. G*′ > *G*′′.^49,50^

Oscillatory shear rheometry also facilitates DMA type measurements where *G*′ and *G*′′ are recorded as a function of temperature or deformation rate (frequency), which allows to determine, *e.g.*, the *T*_g_ of a material from the peak in *G*′′ or the loss tangent tan *δ* = *G*′′/*G*′ (Fig. 7).^47,51,52^ For example, Gomez *et al.* have used oscillatory shear rheometry to determine the *T*_g_ of a wide range of conjugated polymers, which allowed to develop an empirical model for predicting the *T*_g_ based on the makeup of the repeat unit.^51^

### 3.2. Thin-film measurement techniques

Thin-film techniques are advantageous because they only require small amounts of material per sample (1–2 mg) and allow to measure the type of geometry (thin films; thickness *d* ≤ 1 μm; Table 1) that are relevant for most devices (*cf.*Fig. 1). Hence, the mechanical properties of conjugated polymers are often studied with thin-film techniques. However, a direct comparison of values measured with various methods should be done with care since different deformation modes (*e.g.* tensile deformation *vs.* nanoindentation) as well as the need for specific sample geometries and thicknesses can lead to disparate results as recently reported for, *e.g.*, P3ATs.^53^

#### 3.2.1. Force microscopy

**Fig. 8:**
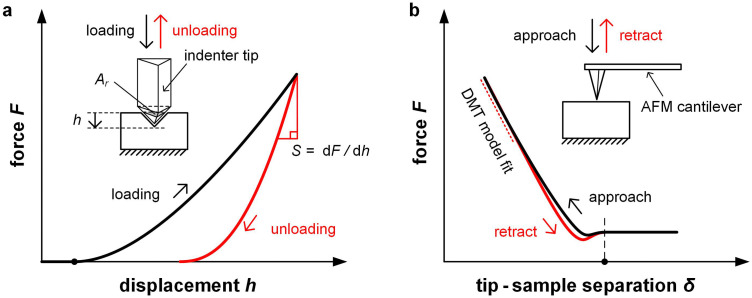
Force microscopy techniques. (a) Nanoindentation involves indentation of a polymer film with an indenter tip, followed by recording the unloading force curve from which the stiffness *S* can be determined; and (b) atomic force microscopy (AFM) can be used to measure the repulsive force between the cantilever tip and film surface, which can be fitted with the Derjaguin–Muller–Toporov (DMT) model.

Nanoindentation and atomic force microscopy (AFM) allow to evaluate the local mechanical properties of a flat sample by recording force–displacement curves as a function of position (Fig. 8).^54^ Both techniques provide information about the reduced elastic modulus *E*_r_ which (provided the probe tip is much stiffer than the sample) is related to the elastic modulus *E* according to:^54,55^3
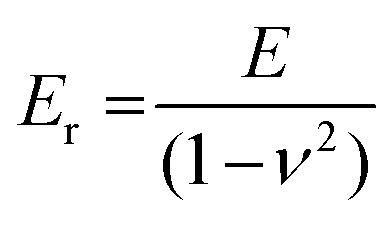
where *ν* is the Poisson's ratio of the material, which must be determined by other means. A perfectly incompressible material yields a Poisson's ratio of *ν* = 0.5; for P3HT a value of *ν* = 0.35 has been predicted.^56^ The shear and tensile elastic modulus (Young's modulus) are related according to:^57^

4 *E* = 2*G*(1 + *ν*)

In case of nanoindentation, an indentation tip with a conical, triangular pyramidal (Berkovich) or cylindrical shape applies a variable load and as a result penetrates the sample causing deformation. The force *F* is recorded as a function of depth *h* during loading-unloading cycles (strain- or force-controlled mode), whose frequency can be varied enabling dynamic measurements similar to DMA (see Section 3.1.2). For relatively soft materials such as polymers, it is important that the maximum indentation depth does not exceed approximately one tenth of the total film thickness (typically *d* > 1 μm) so that the stiffness of the underlying substrate does not influence the measurement. The stiffness *S* = d*F*/d*h* of an elastic material can be extracted by the Oliver–Pharr method from the first derivative of the part of the force–displacement curve that is recorded at the beginning of the unloading cycle (Fig. 8) and is related to *E*_r_ according to:^55^5
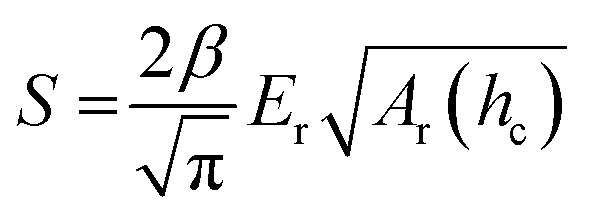
where *β* is a geometrical constant (*β* ≈ 1.05 for a Berkovich tip) and *A*_r_(*h*_c_) the projected area of the indentation at the contact depth *h*_c_. While the Oliver–Pharr method has been used for a wide range of polymers, it should be noted that *E*_r_ of viscoelastic materials is typically overestimated.^58,59^ An alternative method instead determines the creep compliance from the loading cycle,^58^ from which the elastic modulus can be obtained yielding values that are in better agreement with other techniques, as recently shown for P3HT.^60^ In addition to the elastic modulus, nanoindentation can provide information about (1) the hardness *H* = *F*_max_/*A*_r_(*h*_c_) of a sample, which is obtained from the maximum applied load *F*_max_, and (2) the toughness, which can be inferred from crack formation as a result of indentation.

Unlike nanoindentation, mechanical measurements with AFM only allow to access the linear (elastic) deformation regime since the interaction between the AFM tip and sample surface occurs through adhesive/repulsive forces rather than prolonged contact. Hence, only *E*_r_ can be extracted. Typically, the repulsive force between sample and tip is recorded as a function of the cantilever displacement, and *E*_r_ is obtained by evaluating the force–displacement curve with the Derjaguin–Muller–Toporov (DMT) model (Fig. 8):^61^6
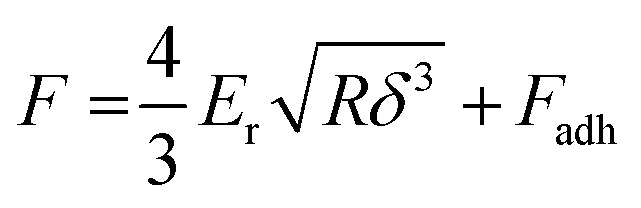
where *R* is the radius of the probe tip, *δ* is the tip-sample separation and *F*_adh_ is the adhesion force. Again, it is important that the film is sufficiently thick to minimize any influence from the typically much stiffer substrate.

#### 3.2.2. Film-on-water tensile testing

**Fig. 9:**
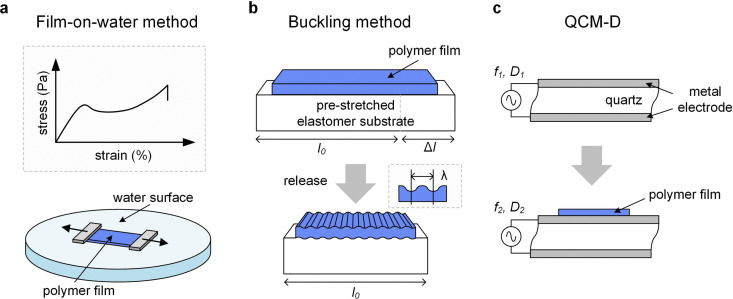
Thin-film techniques. (a) Film-on-water tensile testing can be used to record stress–strain curves; (b) the buckling method involves the release of a pre-stretched elastic substrate with original length *l*_0_ and initial deformation by Δ*l* resulting in buckling of the polymer film with a stiffness-depending wavelength *λ*, and (c) quartz crystal microbalance with dissipation monitoring (QCM-D) records the change in oscillation frequency Δ*f* = *f*_2_ − *f*_1_ and energy dissipation Δ*D* = *D*_2_ − *D*_1_ of a quartz crystal upon deposition of a polymer film.

This technique resembles tensile deformation of free-standing samples and entails stretching of thin polymer films (*d* ≤ 100 nm) deposited on a water–air interface (Fig. 9).^54^ Measurements are usually carried out at room temperature, or slightly above, *e.g.* up to 50 °C in case of PDPP-3T.^62^ Thin films are deposited on a rigid substrate coated with a water-soluble sacrificial layer made of, *e.g.*, PSS, patterned and then removed by floating on water. PDMS clamps grip the film suspended on water and the force, which is required to move the clamps apart to stretch the sample with a certain strain rate, is recorded. A stress–strain curve comparable to that of bulk measurements is obtained, from which *E*, *σ*_yield_ and *ε*_break_ can be obtained (see Section 3.1.1). Moreover, monitoring of the sample with a camera during deformation provides information about the crack onset strain *ε*_crack_ (similar to *ε*_break_ for a classical tensile deformation experiment; see Section 3.1.1).

The high surface tension and low viscosity of the water surface results in a pseudo free standing specimen, which helps to minimize any influence from the substrate. However, the sample can be affected by the uptake of water. A comparison of tensile deformation of (1) free-standing films of regio-regular P3HT and (2) samples supported by water (*d* ≈ 80 nm) revealed a slight reduction in elastic modulus but increase in *ε*_break_ from less than 50% to more than 100% in case of the latter, which could be explained with plasticization by water despite the hydrophobic nature of the polymer.^53^

#### 3.2.3. Buckling method

The buckling method has been widely used to characterize the elastic modulus of conjugated polymer films.^63,64^ A thin polymer film (*d* ≈ 100 nm) is deposited on a pre-strained (*ε* ≈ 10%) elastic substrate (*d* > 1 mm) made of, *e.g.*, PDMS (Fig. 9). Release of the pre-strain allows the substrate to contract, resulting in compression of the polymer film, which buckles provided its stiffness is sufficiently high. Buckling introduces a periodic pattern of wrinkles with a wavelength *λ* that is related to the reduced elastic modulus of the film and substrate, *E*_r_ and *E*^sub^_r_, according to:^64^7
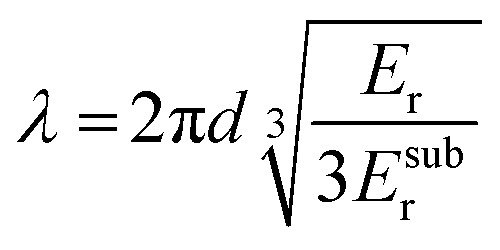
which is valid for small pre-strains and *E*_r_/*E*^sub^_r_ ≫ 1. Optical microscopy or laser diffraction can be used to determine *λ*, which allows to calculate *E*_r_ (see eqn (3)).

A variation of the buckling method involves cyclic tensile deformation of a film on an unstrained substrate with the maximum strain increasing for each cycle. Buckling occurs during the relaxation step once the film starts to undergo plastic deformation because the maximum strain exceeds *ε*_yield_, thus providing information about the latter.^65^ Instead, if tensile deformation is allowed to proceed, fracture of the polymer ultimately occurs, which can be used to determine *ε*_crack_. A disadvantage of the buckling method is the limited control over the deformation rate. A comparison of the film-on-water and buckling method yielded a higher elastic modulus for P3HT in case of the latter, which was explained with an at least 100 times higher strain rate in case of the buckling method as well as differences in the mode of deformation (tension *vs.* compression).^66^ Moreover, buckling experiments are typically carried out at room temperature.

#### 3.2.4. Quartz crystal microbalance

A quartz crystal microbalance (QCM and when combined with dissipation monitoring QCM-D) allows to monitor sorption and desorption processes. When coupled with an electrochemical cell (EQCM/EQCM-D), it is possible to study swelling and ion uptake by conjugated polymer films during electrochemical doping (Table 1).^67,68^ A polymer film is coated onto a quartz crystal and changes of its oscillation frequency Δ*f* and energy dissipation Δ*D* are recorded (Fig. 9), which can be translated into changes in mass (or film thickness). In case of an elastic material, for which energy dissipation is low, the mass change Δ*m* during a sorption process is given by the Sauerbrey equation:^68^8
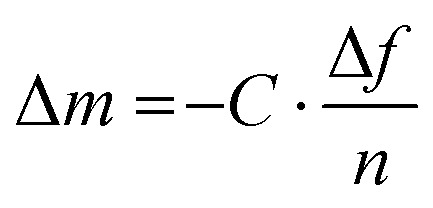
where *C* is the mass sensitivity constant of the sensor and *n* is the number of the harmonic that experiences the frequency shift Δ*f*.

Instead, to describe the response of a sensor coated with a viscoelastic material, for which energy dissipation must be considered, a Kelvin–Voigt model can be used with a complex shear modulus given by:^68^

9 G* = *G*′ + *iG*′′ = *ζ* + *i*2π*fη*

where *ζ* is the elasticity and *η* the viscosity. For instance, EQCM-D in combination with a Kelvin–Voigt model has been used to monitor the amount of mass uptake and swelling of p(g_3_2T-TT) with triethylene glycol side chains (see Fig. 2 for chemical structure) upon electrochemical oxidation, which was found to significantly depend on the ionic strength of the NaCl based aqueous electrolyte.^69^ In another study, EQCM-D was used to show that both passive and active swelling increase with the length and grafting density of oligoether side chains (see Section 6).^70^

### 3.3. Molecular dynamics simulations

Molecular dynamics simulations can be used to investigate the response of conjugated polymers to strain. First a periodic computational box is created that contains a set of polymer chains as well as other species such as solvent, dopant or acceptor molecules. It is usually necessary to restrict the length of polymer chains and the overall number of molecules in order to limit the computational complexity. A force–field is selected, *i.e.* a set of mathematical equations that describe the interactions within and between molecules such as chemical bonds and van der Waals forces. Coarse-grained potentials can be used that treat polymer chains not as an assembly of atoms but beads that interact with each other, thus gaining computational efficiency at the expense of atomistic detail. The molecules in the simulation box are allowed to relax for a certain period of time, followed by uniaxial tensile deformation of the simulation box, which allows to compute the stress–strain curve. Deformation is typically carried out with a high constant strain rate such as *ε̇* = 1 ns^−1^, which implies that the response in the glassy regime is modelled, resulting in a high Young's modulus of several GPa, as predicted by molecular dynamics simulations for, *e.g.*, polythiophenes with both alkyl and oligoether side chains, *i.e.* P3HT and p(g_4_2T-T).^39,56,71^

Molecular dynamics simulations can be used to estimate the *T*_g_. For instance, the density of the simulation box as a function of temperature can provide information about the *T*_g_ at which the linear expansion coefficient changes, leading to a more rapid decrease in density upon further heating.^72^ Moreover, molecular dynamics simulations can be used to study the temperature dependence of thermal vibrations of different building blocks such as aromatic rings and side chains, which provides information about local relaxation dynamics and thus the *T*_g_.^73^

## 4. Rheological and mechanical properties of conjugated polymers

Conjugated polymers follow the same scaling laws that govern other types of polymers. Polymeric materials are able to dissipate stress *via* conformational changes that can occur on a wide range of length scales, from the size of single functional groups, side chains or repeat units to entire polymer chains, provided that the time available for relaxation is sufficiently long. This yields a complex viscoelastic behavior with rheological and mechanical properties being both time/frequency/rate and temperature dependent.

### 4.1. Viscoelastic properties of disordered polymers

The classical description of an *amorphous* polymer considers three principal regions, the glassy, rubbery and viscous regime (Fig. 7), which occur in different time (frequency) or temperature domains, with time and temperature being interchangeable according to the time-temperature superposition principle.^57^ Oscillatory shear rheometry of, *e.g.*, P3ATs has confirmed that the same principle is readily applicable to conjugated polymers (Fig. 7).^47^

In the *glassy* regime, polymer chains are unable to relax on a global scale. The conformation of the polymer is “frozen in” on the experimental timescale and the material is brittle, characterized by a large *G*′ (Fig. 7) but low *ε*_break_. However, local relaxation processes can occur such as relaxation of the side chains of conjugated polymers, which are only frozen in at low temperatures (*e.g.* below a β-relaxation temperature *T*_β_; see Fig. 7). Local relaxation allows glassy polymers to absorb impact energy even below *T*_g_ resulting in some degree of impact toughness.^46^

**Fig. 10:**
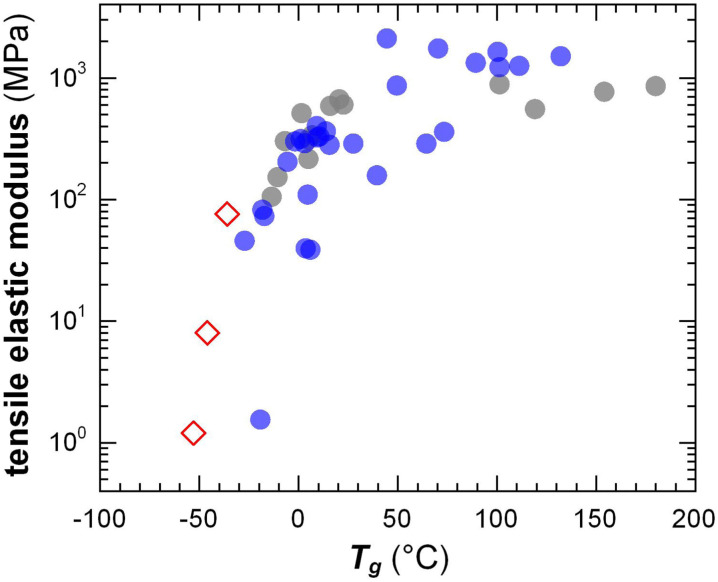
Tensile elastic modulus close to room temperature *vs.* the *T*_g_ of conjugated polymers with oligoether (open diamonds) and alkyl side chains (filled circles). Figure adapted with permission from ref. 79; Copyright 2023 (CC-BY), American Chemical Society, with values from tensile testing or DMA (grey),^38,80–83^ oscillatory shear rheometry (blue; converted using eqn (4) and assuming *υ* = 0.5)^47,51^ and one datapoint added from ref. 84.

The transition from the *glassy* to the *rubbery* regime (or to the viscous regime if the polymer is not entangled) occurs around the *T*_g_, marked by a strong drop in *G*′ and a peak in *G*′′ as well as tan *δ* = *G*′′/*G*′ (note that the *T*_g_ increases with heating/cooling rate). The *T*_g_ of conjugated polymers can be predicted despite strong variations in the makeup of the conjugated (aromatic) backbone as well as the side-chain length and density by considering the difference in mobility of conjugated and non-conjugated atoms.^51^ A more refined prediction of the *T*_g_ could be obtained with a machine-learning model that considered the side-chain fraction, the number of isolated, fused, and bridged aromatic rings, the number of halogen atoms, and the number of double and triple bonds that are not part of aromatic rings.^73^ Generally, a decrease in side-chain fraction correlates with an increase in *T*_g_.^52,74^ For instance, a decrease in the side-chain fraction through the incorporation of unsubstituted aromatic units leads to an increase in *T*_g_, as reported for, *e.g.*, DPP-based copolymers whose backbone comprised varying numbers of thiophene rings or fused thiophene units such as thienothiophene.^75^ Instead, an increase in the side-chain length without altering the makeup of the backbone tends to result in a reduction in *T*_g_, as observed for, *e.g.*, P3ATs.^76^ The *T*_g_ of a (conjugated) polymer determines its mechanical properties around room temperature, *i.e.* the temperature range where most conjugated polymers are used (*cf.* Section 7). Hence, a change in side-chain fraction can be used to adjust the elastic modulus.^77^ Polymers with a low *T*_g_ < 0 °C such as polythiophenes with oligoether or long alkyl side chains are soft at room temperature with a tensile elastic modulus *E*′ as little as 1–10 MPa. Instead, semi-flexible conjugated polymers with a *T*_g_ > 50 °C feature *E*′ ≈ 1 GPa (Fig. 10), while rigid ladder type polymers feature even higher values of, *e.g.*, 8 GPa in case of poly(benzimidazobenzophenanthroline) (BBL) measured with nanoindentation,^78^ likely because of the absence of side chains.

**Fig. 11:**
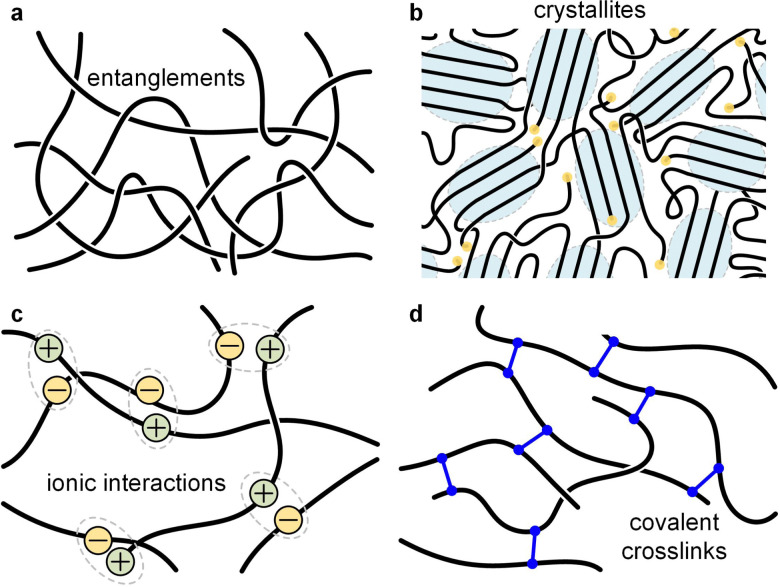
Illustration of different types of network points. (a) Entanglements; (b) crystallites; (c) ionic interactions; and (d) covalent crosslinks.

In the rubbery regime longer sections of polymer chains are able to relax, *i.e.* they can adopt a new conformation and thus dissipate stress. Polymer chains are held in place by entanglements provided they are sufficiently long, which is the case if *M*_n_ is larger than the entanglement molecular weight *M*_e_, *i.e.* the minimum molecular weight required for polymer chains to entangle (*M*_e_ ≈ 25 kg mol^−1^ for P3HT; see Fig. 11a for schematic of an entangled melt).^13^ Entanglements only persist at timescales less than the time required by chains to disentangle.

In addition to entanglements, polymer chains can form a network *via* chemical crosslinks, which can be covalent bonds or strong secondary interactions, such as ionic crosslinks. The plateau modulus of a polymer network melt or solution (see Fig. 7) is given by:^57^10
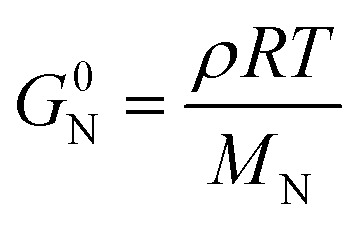
where *ρ* is the density at temperature *T* and *R* = 8.314 J K^−1^ mol^−1^ is the universal gas constant. The molecular weight *M*_N_ between network points, *e.g.* entanglements (in which case *M*_N_ = *M*_e_) and/or crosslinks, depends on the chain conformation and therefore *G*^0^_N_ of an isotropic melt can be predicted for both flexible and semiflexible (conjugated) polymers.^85^ However, the melt or solution of a conjugated polymer can feature nematic (liquid-crystalline) order, which results in less chain entanglements (larger *M*_e_) and therefore a lower *G*^0^_N_ compared to the isotropic state.^86^

In the *viscous* regime, polymer chains are able to relax freely, unimpaired by entanglements since polymer chains have sufficient time to disentangle and adopt a new conformation. The cross-over time between the rubbery and viscous regime is given by the disentanglement time, meaning that (1) low molecular weight polymers, which do not entangle, only show a glassy and viscous regime, and (2) chemically crosslinked materials cannot disentangle, *i.e.* the “rubber plateau” continues to exist also at higher temperatures (Fig. 7) and longer times/lower frequencies.

### 4.2. Polymer networks

Most polymer-based materials can be thought of as an assembly of macromolecules that are connected in various ways, thus forming a network that traverses the material. The IUPAC gold book defines a polymer network as “*a highly ramified macromolecule in which essentially each constitutional unit is connected to each other constitutional unit and to the macroscopic phase boundary by many permanent paths through the macromolecule*”.^87^ Connections, or network points, can be of covalent or physical nature, with the latter including crystallites, glassy domains, (trapped) entanglements as well as different types of secondary interactions such as hydrogen bonds, ionic interactions and π-stacking (Fig. 11). According to eqn (10), the rubber plateau modulus *G*^0^_N_ increases with the number of crosslinks, which reduce the molecular weight between network points. Covalent network points, or *crosslinks*, tend to be permanent and have been used to improve the stability of conjugated polymer films, which is typically achieved through the incorporation of side chains that comprise crosslinkable moieties such as acrylate, azide, vinyl or oxetane groups.^88,89^ Moreover, there are dynamic covalent bonds that reversibly associate or dissociate depending on stimuli such as temperature, pH, *etc.* Dynamic covalent bonds receive considerable attention for the design of stimuli responsive materials^90^ but are, so far, only occasionally explored in the context of conjugated polymers.^91^

Physical network points are mostly dynamic since they tend to disappear when certain stimuli are applied, such as, chiefly, temperature. For example, crystallites (or more generally ordered domains) disappear upon heating above *T*_m_, hydrogen bonds dissociate above a certain critical temperature, and entanglements of a non-crosslinked polymer melt can disappear when the material experiences elongational deformation.

Since many conjugated polymers π-stack at least to some extent, ordered domains are typically present, which reinforce the material between *T*_g_ and *T*_m_, resulting in a storage modulus of 100 MPa to 1 GPa depending on the degree of order. For example, regio-random P3HT, which cannot order, features a low *G*′ of not more than 10 MPa at room temperature, while crystallites in case of regio-regular P3HT lead to a much higher *G*′ of about 100 MPa despite a similar *T*_g_.^47^ Semi-crystalline polymers such as regio-regular P3HT with a low *T*_g_ tend to be ductile at room temperature with *ε*_break_ ≫ 100% provided the molecular weight is high enough (and the right processing technique is selected; *cf.* spin-coating *vs.* spray-coating^92^) so that tie chains and trapped entanglements can connect nearby crystallites (see Fig. 11b).^13^ Instead, conjugated polymers with a high *T*_g_ are usually reported to be brittle since most measurements are carried out at room temperature^76^ and would only become ductile if deformed at elevated temperatures (provided the molecular weight is sufficiently high).

### 4.3. Multi-component systems

Conjugated polymers can be combined with many different types of materials such as small-molecular additives, reinforcing agents or other polymers, to form various multi-component systems. While the resulting mixtures, composites or blends are usually created to modify the optical and electrical properties of the semiconductor, in many cases a significant influence on the mechanical properties is observed.

**Fig. 12:**
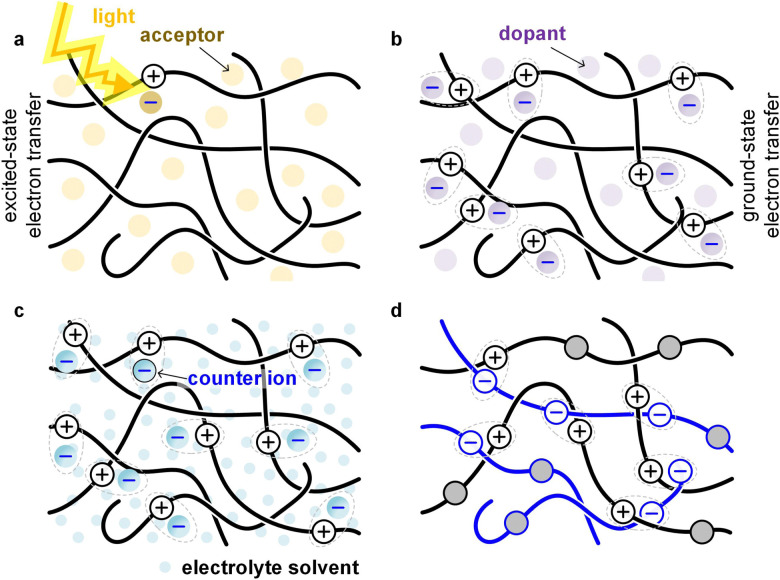
Illustration of different types of multi-component systems that involve electron transfer. (a) Bulk-heterojunction blend of a donor polymer and an acceptor that undergo excited-state electron transfer; (b) mixture of a donor polymer and a p-dopant that undergo ground-state electron transfer; (c) electrochemically oxidized donor polymer that takes up anions and electrolyte solvent; and (d) blend of a p- and n-type polymer that undergo ground-state electron transfer (all-polymer blends that undergo excited-state electron transfer are omitted).

A common type of multi-component system within the field of organic electronics are bulk-heterojunction blends that form the active layer of OPV devices. A donor polymer is mixed with a small-molecular or polymeric acceptor, which – once the polymer absorbs light – undergo excited-state electron transfer (Fig. 12). The acceptor can cause embrittlement of the bulk-heterojunction blend,^93^ for example because of the presence of acceptor-rich domains with poor mechanical connectivity. Besides the distribution and size of domains, the connectivity in donor:acceptor bulk-heterojunction blends depends on the molecular weight of the polymer located in donor-rich as well as mixed domains, which influences both the electrical properties (solar cell efficiency) as well as the mechanical properties (fracture energy).^45^

Small-molecular additives can be added that at low concentrations function as, *e.g.*, a plasticizer that decreases *T*_g_, at larger concentrations swell the polymer and at high concentrations function as a solvent. Additives can interact with the polymer. A common way to plasticize polymers that form hydrogen bonds is the addition of polar molecules such as water, which decrease the interactions between polymer chains by forming hydrogen bonds with the polymer instead. Another common type of additive is a small-molecular dopant that undergoes ground-state electron transfer with a polymer, resulting in a counterion that balances the charge (polaron) that has been created on the polymer (Fig. 12; see Section 5). Alternatively, the polymer is electrochemically oxidized and takes up counterions as well as solvent molecules from an electrolyte solution (Fig. 12; see Section 6).

Other types of multicomponent systems are blends (or AB copolymers) of two or more polymers; a conjugated polymer can be combined with another conjugated polymer, an insulating polymer or a polyelectrolyte. Blends of two polymers can – depending on their energy levels – undergo ground-state or excited-state electron transfer (Fig. 12), resulting in a chemically doped material or a bulk-heterojunction blend for OPV devices, respectively. The second polymer can also be an insulator, *e.g.* polyethylene or polystyrene, whose purpose is to adjust the rheological and mechanical properties of the conjugated polymer.^94^ Moreover, the second polymer can be a polyelectrolyte, *e.g.* PSS, which then provides the counterions for electrical charges (polarons) on the conjugated polymer. The most widely studied conjugated polymer:polyelectrolyte complex is PEDOT:PSS,^6^ and numerous ways to modify its mechanical properties have been explored, including plasticization and blending with other polymers.^95^

The conjugated polymer can also form a composite with a reinforcing agent such as cellulose nanocrystals (CNC) or cellulose nanofibrils (CNF), a carbon allotrope such as graphene or carbon nanotubes (CNTs) as well as other 2D materials such as MXenes. Carbon allotropes are usually added to modify the electrical properties of conjugated polymers,^96^ but it can be anticipated that they also act as a reinforcing agent, which is commonly observed for nanocomposites with commodity polymers.^97^ Nanocellulose, instead, is more widely used to modify the rheological and mechanical properties of conjugated polymers,^98^*e.g.* for wet-spinning of fibers composed of CNF and PEDOT:PSS,^99^ but can also enhance the ionic mobility (see Section 7.4).^79^

### 4.4. Anisotropy and orientation

Flexible polymer chains adopt a random-coil conformation to maximize entropy but can align in flow fields or as a result of mechanical force, *e.g.* a tensile or shear force (*cf.* rubbing). Alignment of a polymer can only be achieved above the *T*_g_, where chains can change their conformation. In case of an amorphous polymer, chains are also prone to relax and hence orientation is only preserved if the aligned material is quickly cooled to a temperature below *T*_g_. Alignment of semicrystalline polymers, instead, can be carried out below *T*_m_ (ideally close to *T*_m_ since slip of polymer chains in crystallites is then possible) and results in aligned polymer chains that retain their orientation because of the presence of ordered domains (crystallites). If the polymer matrix contains a second material, *e.g.* CNF or CNTs, alignment of the nanomaterial can occur. Materials that are aligned to at least some degree tend to feature anisotropic optical, electrical and mechanical properties, with significantly increased electrical conductivity and elastic modulus along the direction of chain alignment.

Thin films of conjugated polymers can be uniaxially aligned through tensile drawing on a stretchable substrate and by shear, *e.g.* through rubbing of solid films on a rigid substrate.^100,101^ Similarly, orientation of free-standing bulk samples can be achieved by solid-state tensile drawing that is terminated prior to fracture or through compression molding of a solid material.^102^ Fiber spinning of conjugated polymers paired with solid-state drawing, for example, tends to yield filaments with a high degree of uniaxial alignment along the fiber axis, which is essential for achieving a high stiffness and stress at break *σ*_break_ (tensile strength).^37^ How the properties along the two directions perpendicular to the direction of chain alignment are affected depends on the secondary interactions between chains. Strong interactions such as hydrogen bonding or π-stacking tend to strengthen a material perpendicular to the direction of chain alignment and may even enhance charge transport, as observed for solid-state pressed P3HT, which features the highest charge-carrier mobility along the π-stacking direction.^103^

## 5. Impact of chemical doping on the mechanical properties

Conjugated polymers can exist as neutral semiconductors or they can be in an oxidized or reduced state, which emerge as a result of chemical or electrochemical doping. Alternatively, the oxidized or reduced forms are obtained directly from certain polymerization routes such as oxidative polymerization, yielding materials such as PEDOT:PSS and PBFDO (see Section 2). The electrical as well as mechanical properties change with the degree of oxidation or reduction of the polymer and hence can be controlled *via* doping.

### 5.1. Chemical doping

Chemical doping is a widely used tool for modulating the number of charge carriers *n* and work function of organic semiconductor films, resulting in an increase in electrical conductivity according to:11
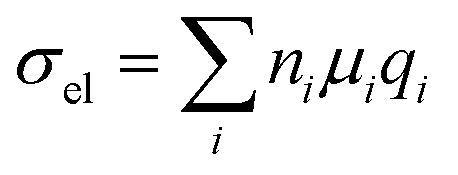
where *n*_*i*_ is the number, *μ*_*i*_ the mobility and *q*_*i*_ the charge of (free) charge-carriers of type *i*. The two most common doping mechanisms are redox doping and acid–base doping,^2,96^ or a combination of the two, which occurs in case of certain Lewis acid dopants.^104^

Redox doping entails the addition of an oxidizing or reducing agent (the dopant) that exchanges one or several electrons with the conjugated polymer resulting in either p- or n-doping. The polymer donates or accepts electrons resulting in polarons on the polymer backbone, whose charge is balanced by the ionized dopant molecules that remain as counterions. In case of acid–base doping, the dopant (*e.g.* a protonic acid or N-DMBI; see Fig. 2 for chemical structure) and the semiconductor exchange a proton (H^+^)^105,106^ or hydride (H^−^)^105^ resulting in a p- or n-doped material, respectively.

Doped films can be prepared by a variety of different processing methods, such as co- and sequential processing. Co-processing involves mixing of the conjugated polymer and dopant, *e.g. via* dissolution in a common solvent, followed by spin coating, drop casting, fiber spinning, *etc.* Sequential processing, instead, entails the preparation of neat polymer films followed by exposure of the solidified material to a dopant solution or vapor. Another variant is ion-exchange doping, where a semiconductor film is exposed to a strong oxidizing (or reducing) agent that is dissolved in an electrolyte solution. Oxidation of the film is followed by exchange of the dopant counterions with alternative ions provided by the electrolyte solution.^107,108^ For an in-depth discussion of various doping mechanisms and methods, we refer the reader to two recent reviews.^2,109^

If a high degree of control over the microstructure of a sample is required, sequential doping is typically the preferred method. However, sequential processing of thick films or bulk samples, which are needed for certain mechanical measurements (see Section 3) as well as applications that require free-standing architectures (see Section 7), can result in inhomogeneous doping throughout the material because dopant molecules must diffuse into the sample.^110^ To achieve a homogeneous distribution of the dopant, porous structures such as foams can be used that ease the ingression of dopant molecules into the material.^111^ Alternatively, some combinations of polymer and dopant can be co-processed into bulk samples, such as PANI and DBSA, where the dodecyl chains of the dopant impart melt-processability.^21^

Doping can strongly alter the nano- and microstructure of conjugated polymers, which can significantly influence electronic charge transport but also alter the mechanical properties of the semiconductor through a range of effects including plasticization, ionic crosslinks, planarization of the conjugated backbone and a change in the degree of order (π-stacking). A further intriguing avenue is the use of doping reactions or counterion exchange for the preparation of organogels, hydrogels or coacervates. For example, conjugated polymers (including conjugated polyelectrolytes) can form networks in solution through ground-state electron transfer^112^ or *via* ionic crosslinks that involve multivalent acids such as phytic acid (see Fig. 2 for chemical structure),^113^ polyelectrolytes (*cf.* PEDOT:PSS), ionic liquids^114^ or salts that comprise multivalent ions.^115^ A recent example involved the formation of a complex composed of an anionic conjugated polyelectrolyte – a polythiophene with both hexyl and hexyl sulfonate side chains – and a cationic bottlebrush polyelectrolyte. Ionic interactions resulted in a coacervate that after drying yielded a soft material with a low *E* = 0.7 MPa but *ε*_break_ = 430%, which upon doping with H-TFSI turned into an elastic conductor with a lower *E* = 0.2 MPa and *ε*_break_ = 94%.^116^

### 5.2. Low doping regime

**Fig. 13:**
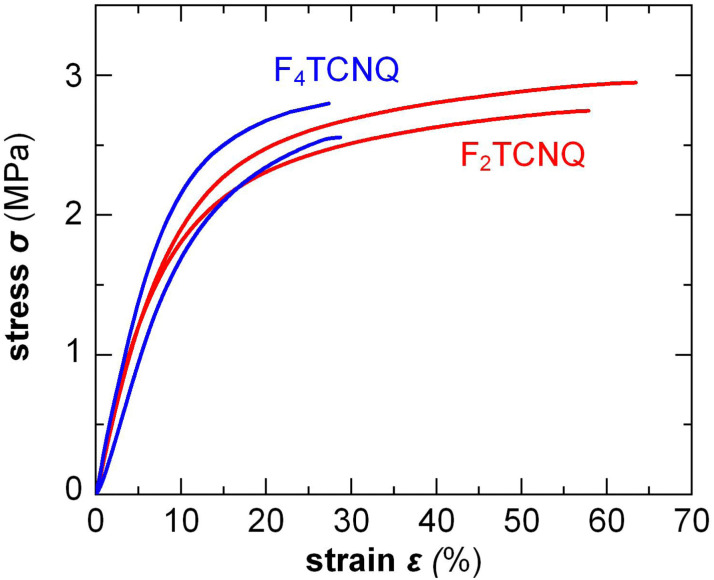
Stress–strain curves measured by tensile deformation of free-standing p(g_4_2T-T) films co-processed with 3 mol% F_4_TCNQ or 6 mol% F_2_TCNQ, resulting in a similar oxidation level of 5.7 and 6.4%, respectively; Figure adapted with permission from ref. 39; Copyright 2022 (CC-BY), The Royal Society of Chemistry.

Small amounts of a dopant of not more than a few mol% (with respect to the polymer repeat unit) can be added to organic semiconductors to fill traps, adjust the work function, raise the electrical conductivity but also to modify the nanostructure and texture of the host.^2^ Accordingly, changes in mechanical properties can be anticipated. For example, Mun *et al.* studied the impact of 0.5–2 wt% F_4_TCNQ on PDPP-TT (see Fig. 2 for chemical structure) and found that co-processing reduces the degree of order of the polymer, resulting in films with a higher ductility, as evidenced by an increase in *ε*_crack_ from 20 to 75%.^117^ In case of p(g_4_2T-T), instead, co-processing with 3 mol% F_4_TCNQ (oxidation level 5.7%) or 4 mol% H-TFSI increased the order of the polymer, which caused embrittlement, *i.e. ε*_break_ decreased from about 160% to less than 30%.^39,118^ Interestingly, doping with F_2_TCNQ retained the ductility to a greater extent for a similar oxidation level of about 6.4%, as evidenced by a similar *E* = 31 MPa but higher *ε*_break_ ≈ 60% (Fig. 13).^39^ Evidently, the choice of dopant is important for realizing ductile conducting polymers. Moreover, multivalent counterions can improve the resistance towards dopant diffusion, as recently observed for, *e.g.*, the naphthalenediimide (NDI) based copolymer P(NDI2OD-T2) (see Fig. 2) that was n-doped with a derivative comprising four covalently linked DMBI-H moieties.^119^ Similarly, blends of a p- and n-type polymer such as p(g_4_2T-T) and BBL, where each polymer chain can donate or accept several electrons, are characterized by a high degree of stability.^120^ It can be anticipated that multivalent counterions or all-polymer materials can yield materials with more stable electrical as well as mechanical properties.

### 5.3. High doping regime

**Fig. 14:**
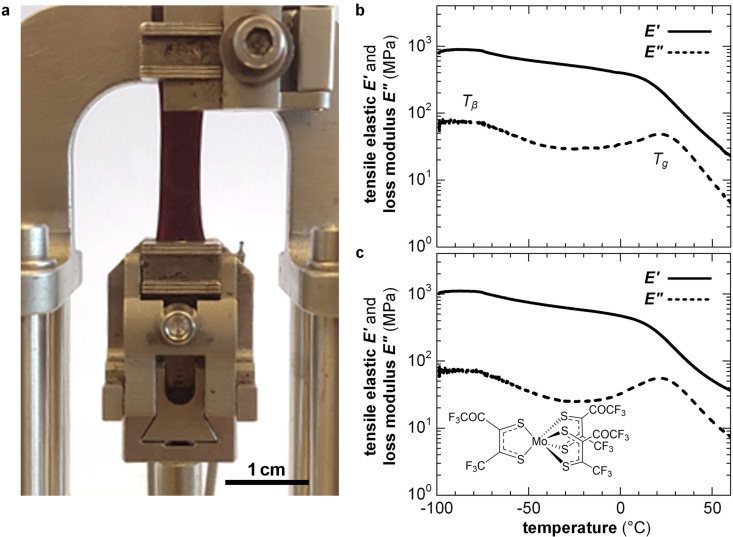
(a) A tensile-drawn regio-regular P3HT film clamped in a dynamic mechanical analyzer, and DMA thermograms of isotropic P3HT films (b) prior to doping and (c) after sequential doping with Mo(tfd-COCF_3_)_3_. Figure adapted with permission from ref. 38; Copyright 2019, American Chemical Society.

Highly doped polymers are needed for applications such as thermoelectrics where a high electrical conductivity is imperative (Section 7). To reach a high doping level a considerable amount of dopant of more than 10 mol% must usually be added, resulting in a two-component material. To fully describe the nano- and microstructure of doped conjugated polymers, besides the degree of order, texture and connectivity of the polymer, the distribution of dopant molecules and counterions must be considered. Co-processing of polymer and dopant molecules can cause aggregation and thus the formation of poorly connected and hence brittle films. For example, solid-state pressing of P3HT blended with 20 mol% ethylbenzene sulfonic acid (EBSA) resulted in an inhomogeneous solid with numerous cracks, while the use of an EBSA-based latent dopant, which is thermally activated subsequent to pressing, gave rise to mechanically robust films.^121^ Instead, sequential processing of P3HT, followed by doping, tends to result in homogeneous films with a high degree of connectivity because the solid-state nanostructure of the polymer develops without the influence of the dopant. For example, free-standing isotropic or tensile-drawn films of P3HT remain mechanically robust upon sequential doping with 9 mol% of the molybdenum dithiolene complex Mo(tfd-COCF_3_)_3_, yielding an *E*′ = 0.5 GPa or 0.4 GPa at 0 °C (compared with 0.6 or 1.1 GPa prior to doping), measured with DMA in tensile mode (Fig. 14).^38^ Doping of isotropic and tensile drawn films did not strongly affect the *T*_g_ ≈ 20 °C and *T*_β_ ≈ −90 °C, indicating that chemical doping can yield conducting materials with a high degree of mechanical robustness and impact toughness (Section 4.1). Interestingly, the modulus of P3HT doped with FeCl_3_ or I_2_ has been found to decrease by a factor of 2–3 upon the application of an electric field of about 1 V mm^−1^, which was only in part due to Joule heating,^122^ suggesting that the mechanical properties of conjugated polymers can be electrically modulated.

In some cases, co-processing of polymer and dopant, or doped polymer and counterion, can result in mechanically robust materials. For example, PANI can be co-dissolved with camphor or aryl sulfonic acids as well as, optionally, insulating polymers such as poly(methyl methacrylate) (PMMA), polystyrene or polypropylene.^21^ Direct ink writing of PANI and DBSA^123^ or dinonylnaphthalene sulfonic acid, polystyrene and fused silica^124^ has been demonstrated, in the latter case resulting in printed filaments with a storage modulus of about 0.2 GPa at room temperature. PANI and DBSA can also be wet-spun into fibers with a diameter below 5 μm that feature an *E* = 30 GPa and *σ*_break_ = 1080 MPa but *ε*_break_ of only 4%.^125^ Similarly, aqueous dispersions of PEDOT:PSS can be used to wet-spin fibers with a diameter of 5–10 μm, an *E* of up to 22 GPa, *σ*_break_ = 550 MPa, *ε*_break_ = 7.5% and an electrical conductivity of 3500 S cm^−1^.^7^ Similar values of *E* = 19.5 GPa and 1600 S cm^−1^ have recently been reported for wet-spun and drawn PBFDO fibers.^9^

**Fig. 15:**
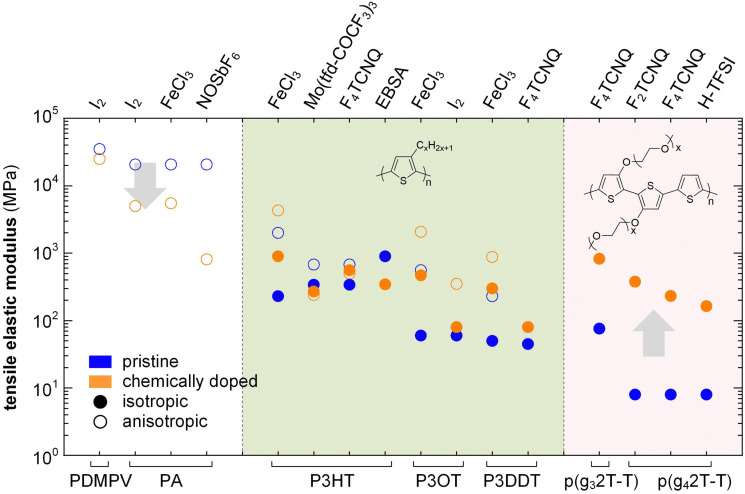
Impact of chemical doping on the tensile storage modulus of PDMPV, PA and various polythiophenes with alkyl or oligoether side chains. Blue circles represent the semiconductor in its neat state, while orange circles are doped polymers; data from ref. 38, 39, 84, 118, 121, 126, 127, 130 and 131.

In the high doping regime materials tend to feature brittle failure with a low *ε*_break_ of typically less than 10%. Chemical doping can both increase or decrease the elastic modulus of conjugated polymers depending on how a particular dopant affects the *T*_g_ and degree of crystalline order. In case of conjugated polymers with *E* > 0.1 GPa, chemical doping only results in a relatively minor change in stiffness, which can both decrease or increase depending on the polymer:dopant pair (Fig. 15). For example, oriented PA tapes and poly(2,5-dimethoxy-*p*-phenylenevinylene) (PDMPV) fibers show a decrease in elastic modulus upon doping, as measured with tensile drawing.^126,127^ Likewise, ion exchange doping slightly reduces the stiffness of PBTTT films, as measured with AFM and nanoindentation,^128^ and regio-regular P3HT, measured with DMA, shows a decrease in elastic modulus when doped with, *e.g.*, EBSA or Mo(tfd-COCF_3_)_3_, likely due to a plasticization effect as evidenced by a concomitant decrease in *T*_g_.^38,121^ Instead, doping of P3HT with F_4_TCNQ or FeCl_3_ tends to result in an increase in elastic modulus, as measured with AFM and tensile drawing,^129,130^ respectively, possibly because of an increase in π-stacking.

A significant increase in *T*_g_ as well as π-stacking is observed upon chemical doping of conjugated polymers with a lower stiffness. For instance, p(g_4_2T-T) with *E* = 8 MPa at room temperature can experience a 29-fold increase in Young's modulus to 232 MPa, along with a change in *T*_g_ from −43 to 3 °C, when doped with 30 mol% F_4_TCNQ (measured with a dynamic mechanical analyzer using static tensile deformation; see Section 3.1.2).^39^ Similarly, the modulus of p(g_4_2T-T) doped with 18 mol% H-TFSI increases 20 times, reaching 164 MPa (Fig. 15).^118^ Shorter triethylene glycol side chains give rise to a stiffer polymer, p(g_3_2T-T), with *E* = 76 MPa, which upon doping with 20 mol% F_4_TCNQ increased to 826 MPa.^84^

### 5.4. Correlation between electrical and mechanical properties

The nano- and microstructure of conjugated polymers tend to impact their electrical as well as mechanical properties and therefore strong correlations exist. The volume fraction and size of ordered domains, which can be crystallites or regions where π-stacking occurs, benefit charge transport and increase the elastic modulus. This behavior is evident when comparing, *e.g.*, regio-regular P3HT and PBTTT, the latter of which featuring a significantly higher *μ* > 0.3 cm^2^ V^−1^ s^−1^ and *E* ≈ 1.8 GPa.^29^ Tie chains improve the connectivity between ordered domains and therefore tend to benefit *μ*. In terms of mechanical properties tie chains are beneficial for achieving a ductile material with a high *ε*_break_ (Section 3). Another important parameter is uniaxial orientation, which enhances both *μ* and *E*′ along the direction of alignment (accompanied by a decrease in the perpendicular direction), as reported for stretch-aligned P3HT.^29^

Similar correlations are observed for chemically doped polymers. For example, both the electrical conductivity and Young's modulus of p(g_4_2T-T) increase in tandem upon doping with F_4_TCNQ.^39^ Doping increases the charge-carrier density but also induces π-stacking and thus enhances the charge-carrier mobility, both of which lead to an increase in the electrical conductivity (see eqn (11)). At the same time, the increase in π-stacking as well as the introduction of Coulomb interactions between polarons and dopant counterions lead to stronger interactions between molecules. As a result, *E* is enhanced, which is the energy per volume stored in a material upon elastic deformation (small strains) and can be understood as the resistance to rearrangement of nearby molecules. Other dopants such as H-TFSI induce π-stacking in the low doping regime, while in the high doping regime π-stacking is disrupted, resulting in a breakdown of the correlation between electrical conductivity and Young's modulus,^118^ which may prove useful for the design of conducting but not overly stiff materials.

Uniaxial orientation tends to result in a strong enhancement of both the electrical conductivity and Young's modulus, which both scale with the degree of orientation. For example, a linear correlation has been reported for oriented P3AT fibers doped with FeCl_3_,^130^ PDMPV fibers doped with I_2_^127^ and PEDOT:PSS fibers.^7^ Wet-spun poly(3-octylthiophene) (P3OT) fibers doped with FeCl_3_ feature values of *E* ≈ 0.5 GPa and an electrical conductivity of 25 S cm^−1^, which increase to 2.2 GPa and 180 S cm^−1^ for a draw down ratio of 5.5.^131^ The approximately linear correlation between the electrical conductivity and Young's modulus in case of doped fibers is also evident when comparing champion values reported for different conjugated polymers.^37^

## 6. Impact of electrochemical doping on the mechanical properties

Electrochemical doping describes the oxidation or reduction of a semiconductor through the transfer of electrons to or from a working electrode, with the resulting charge on the polymer compensated *via* the influx of ions and solvent molecules from a liquid or solid electrolyte that is in contact with a counter electrode (Fig. 12). Hence, the mechanical properties of electrochemically doped polymers depend not only on the change in the structure of the polymer and interactions with counterions, as is the case for chemical doping, but also on polymer–solvent interactions.

The number of transferred electrons depends on the potential that is applied at the electrode and the speed of electrochemical doping is governed by the drift of ions into the semiconductor. The degree of electrochemical doping can be readily altered, or even reversed, by changing the potential that is applied at the working electrode. The accompanied uptake/expulsion of ions and solvent molecules leads to switchable changes in the volume of the polymer, which is widely used for the design of actuators^5^ and can be anticipated to lead to electrochemically mutable mechanical properties, which could be exploited for the design of new types of mechatronic devices.

### 6.1. Electrochemical doping

In case of a hydrophobic conjugated polymer such as P3HT electrochemical doping commences by the formation of an electrostatic double layer of electrolyte ions and polarons at the electrolyte/polymer interface, followed by diffusion of the ion/polaron pair into the polymer.^132^ The diffusion coefficient of ions tends to be low, having a value of 10^−14^ cm^2^ s^−1^ in case of Cl^−^ in a bithiophene–thienothiophene copolymer with alkoxy side chains^70^ and 10^−11^ cm^2^ s^−1^ in case of ClO_4_^−^ in P3HT.^132^ The ingression of ions expands the polymer while the oxidation/reduction of the polymer backbone can significantly alter the nanostructure.

More hydrophilic materials such as PEDOT:PSS and polythiophenes with oligoether side chains are able to take up not only ions but also solvent molecules. Hence, these materials undergo passive swelling, *i.e.* the uptake of solvent molecules from the electrolyte in the absence of any applied electric field, leading to an increase in volume. As a result, ion-conduction pathways are present, *i.e.* the electrolyte is in contact with the conjugated polymer throughout its entire volume, which facilitates the ingression of ions and hence oxidation/reduction of the whole film once an electrochemical potential is applied.

In case of PEDOT:PSS, which is initially electrically conducting, the application of a negative potential at the working electrode reduces the conjugated polymer and cations ingress into the material to compensate the charge of PSS counterions (depletion mode). Instead, a positive (negative) working electrode potential leads to oxidation (reduction) of initially neutral polymers, accompanied by ingression of anions (cations) to balance the generated charges (accumulation mode). The ions that enter the material are accompanied by solvent molecules, and the amount of solvent that is taken up depends on, *e.g.*, the anion size and electrolyte^133^ as well as the ionic strength of the electrolyte.^69^ This so-called active swelling results in an additional increase in volume, which can exceed 100% in case of polythiophenes with oligoether side chains in combination with an aqueous electrolyte^134,135^ and 20 to 60% in case of PEDOT:PSS.^136,137^ The ingression of ions tends to be faster than for apolar polymers with a diffusion coefficient of up to 10^−9^ cm^2^ s^−1^ in case of Cl^−^ in p(g_3_2T-TT).^70^

Electrochemical doping can significantly alter the nanostructure of the material due to swelling of amorphous domains, as well as the oxidation/reduction of the backbone, leading to, *e.g.*, enhanced (or reduced) π-stacking, expansion of the lamellar stacking distance and changes in texture. For example, the backbone of p(g_3_2T) planarizes upon electrochemical oxidation using an aqueous NaCl electrolyte, which leads to a significant increase in π-stacking that results in a high charge-carrier mobility provided that ordered domains are well connected.^138^ Some changes are irreversible, such as the transformation of initially densely packed films into an open network structure upon pronounced active swelling.^69,139,140^

### 6.2. Competition between stiffening and swelling

Changes in mechanical properties of polymers such as polypyrrole (PPy) and PEDOT upon electrochemical oxidation/reduction have been investigated in the context of actuators, devices that convert electrical energy into mechanical energy. Bulk techniques including tensile testing and DMA in combination with an electrochemical cell^141^ as well as E-QCM^142^ have been used to monitor changes in elastic and loss modulus *in situ*.

**Fig. 16:**
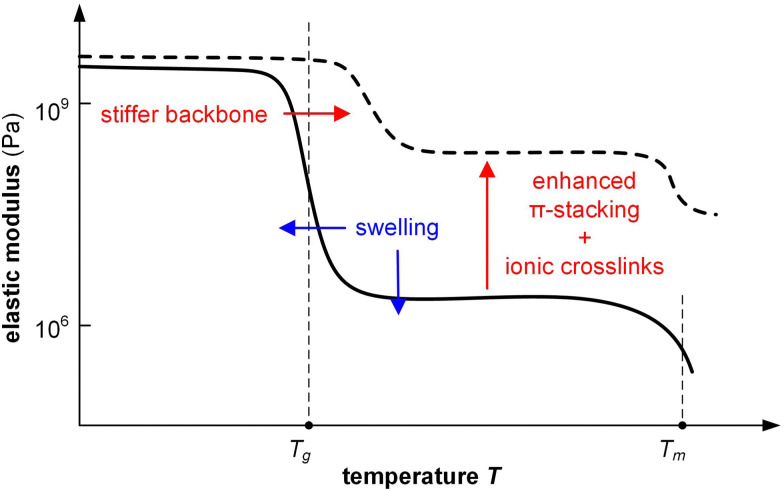
The elastic modulus of a conjugated polymer can change upon doping due to stiffening of the backbone, enhanced π-stacking, the formation of ionic crosslinks through polaron–counterion interactions, and swelling as a result of the uptake of counterions and/or solvent molecules.

Similar to chemical doping, the elastic modulus of a conjugated polymer is affected by a number of processes, including (1) plasticization by counterions and in particular solvent molecules, (2) stiffening of polymer chains due to oxidation (possibly accompanied by a change in ordering), (3) ionic crosslinks between oxidized polymer chains and counterions, and (4) swelling through the uptake of counterions and solvent molecules (Fig. 16).^141^ The relative importance of these in part counteracting effects determines how the elastic modulus of a material changes upon electrochemical doping. For example, a polymer that takes up counterions but repels solvent molecules may show an invariant or even enhanced elastic modulus because ionic crosslinking outweighs plasticization. Instead, a polymer that experiences considerable swelling due to the uptake of solvent molecules that accompany counterions is likely to display a decrease in stiffness.

PPy, widely studied as an actuator material, shows a decrease in *E* from 1 to 0.8 GPa upon oxidation, using aqueous NaPF_6_ as the electrolyte, which was explained with a plasticization effect due to the ingression of PF_6_^−^ anions accompanied by solvent (water) molecules.^143^ Furthermore, the ductility can significantly change with the oxidation level. For instance, PPy electropolymerized in aqueous *p*-toluenesulfonic acid is brittle in its oxidized state, which was explained with ionic crosslinking between charged polymer chains and counterions, but becomes more ductile with *ε*_break_ increasing from 5 to 21% upon electrochemical reduction using aqueous electrolytes such as NaCl with monovalent cations due to plasticization as a result of the ingression of Na^+^.^144^ Instead, the material remained brittle upon reduction using an aqueous MgCl_2_ electrolyte, during which divalent Mg^2+^ cations enter the material. Evidently, the type of counterion can influence the mechanical properties (*cf.*Fig. 13; chemical doping with F_2_TCNQ or F_4_TCNQ). Plasticization due to active swelling has also been inferred in case of electropolymerized P3HT films, where electrochemical oxidation let to a 15% thickness increase due to the ingression of PF_6_^−^ anions and a further 48% increase due to solvent swelling (propylene carbonate), overall resulting in a decrease in elastic modulus.^145^

**Fig. 17:**
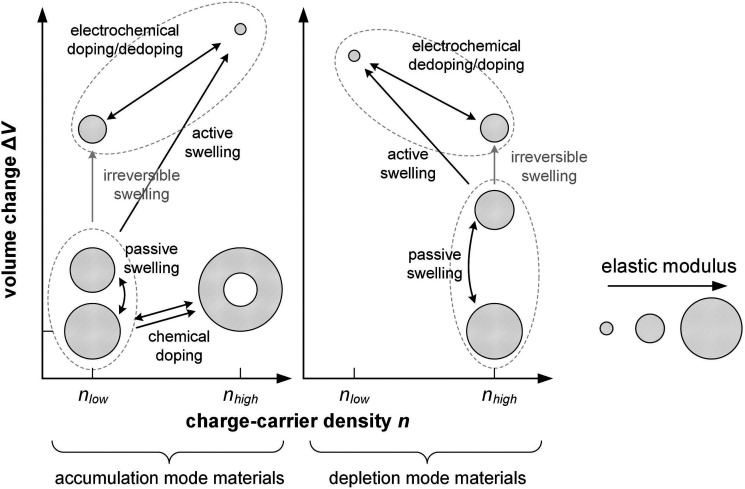
The volume change Δ*V* upon doping can strongly alter the elastic modulus of conjugated polymers and occurs during (1) chemical doping, (2) passive swelling, and active swelling through the uptake of counterions and solvent molecules upon (3) electrochemical doping (accumulation mode) as well as (4) dedoping (depletion mode).

Other materials such as polythiophenes with oligoether side chains such as p(g_3_2T), p(g_4_2T-T) and p(g_3_2T-TT) tend to experience a more significant volume change Δ*V* upon oxidation.^134,135,146,147^ For example, p(g_3_2T) with triethylene glycol side chains turns from a solid material into a gel accompanied by Δ*V* > 1000% during the first oxidation cycle using aqueous KCl, an increase that is not completely reversible because of the partial retention of counterions and solvent molecules during reduction,^134^ as well as permanent structural changes. During subsequent oxidation/reduction cycles, p(g_3_2T) undergoes reversible active swelling by Δ*V* > 200%, which is much larger than passive swelling in the same electrolyte (Fig. 17). A similar polymer with diethylene glycol side chains, p(g_2_2T), only shows a Δ*V* = 27%, which highlights the importance of sufficiently long side chains.^146^ In addition to swelling, p(g_3_2T) experiences an increase in π-stacking upon electrochemical oxidation.^138^ Nevertheless, it can be anticipated that polar polymers such as p(g_3_2T) exhibit a significant reduction in elastic modulus upon electrochemical doping because the large degree of swelling-induced plasticization likely outweighs changes in ordering, consistent with the observed solid-to-gel transition.^134^ PEDOT:PSS experiences considerable passive swelling in aqueous electrolytes, with the degree of swelling depending on the PSS content,^148,149^ as well as in non-aqueous solvents such as acetonitrile and methanol.^137^ The passive swelling ratio of PEDOT:PSS tends to be higher than that of accumulation mode materials (*e.g.*, conjugated polymers without polyelectrolyte) because the protonation/deprotonation of the polyelectrolyte (*i.e.*, PSS) facilitates additional water uptake.^150^ However, PEDOT:PSS undergoes limited active swelling,^150^ which suggests that the material will soften once it is brought in contact with an electrolyte, but exhibits only limited further changes in mechanical properties during reduction/oxidation cycles (see Fig. 17).

A high degree of passive and/or active swelling can limit the stability of electrochemical devices because of irreversible structural changes due to the retention of counterions and solvent as well as gradual delamination of device active layers. Hence, materials that experience minimal swelling upon electrochemical doping are highly sought after. One example is the polythiophene poly(3-(6-hydroxy)hexylthiophene) (P3HHT), which undergoes minimal and hence reversible passive and active swelling with Δ*V* < 10% and therefore recovers its stiffness after each oxidation/reduction cycle.^147^

## 7. Relevance of mechanical properties for different applications

Conjugated polymers are being used for a wide range of applications, from thin-film to bulk devices, each of which has its own requirements with regard to the mechanical properties of the materials.

### 7.1. Thin-film *vs.* bulk devices

The majority of organic electronic devices including OLEDs, OPV devices, OFETs as well as many OTE and electrochemical devices such as OECTs consist of one or several thin layers supported by a substrate that provides mechanical integrity. The various layers and electrodes of thin-film devices can be printed or coated onto the substrate, which usually is a planar surface such as a plastic foil but can also be a non-planar object such as a 3D-printed structure or textile fiber. The rheological properties of the ink solution are important for film formation, both in terms of the nanostructure that can develop during drying as well as the smoothness and homogeneity of the deposited films.

The substrate material, which can be rigid, flexible (bendable) or even stretchable, determines the mechanical properties of the device. Thin-film devices on a rigid substrate do not require organic semiconductor layers with any specific mechanical properties, which arguably is the reason why this type of architecture is often selected for screening of new materials in research laboratories. However, the overly use of rigid substrates tends to divert attention from the mechanical properties that are ultimately required for the design of materials for flexible and/or stretchable electronics.

In contrast, if the substrate is non-rigid, the thin-film layer stack must be able to accommodate the same type of deformation that the substrate (surface) experiences upon bending or stretching, without fracture of any of its constituent layers or loss of adhesion between layers. A substrate with a low stiffness can be realized by selecting a material with a low elastic modulus such as a thermoplastic elastomer or other type of rubber, which will also exhibit a high degree of reversible stretchability.

Alternatively, the thickness of the substrate can be reduced to achieve a low stiffness (see eqn (1), Section 3.1). Plastic foils with a thickness of 0.3 to 3 μm have been used for the design of imperceptible electronics composed of conformable and low-weight devices from OFETs to OLEDs and OPV devices, which can be placed on skin or implanted.^151^ A conjugated polymer layer deposited on top of a low-stiffness substrate will experience compressive and/or tensile stresses upon bending or stretching. The resulting deformation should not exceed *ε*_crack_ (see Section 3.2), which will result in the loss of integrity of the semiconductor layer, especially upon repeated bending or stretching. Most conjugated polymers feature a low yield strain *ε*_yield_ < 10%,^152^ which means that they will undergo plastic deformation when deformed beyond this limit, leading to irreversible changes that may negatively affect the device performance. Hence, depending on the application a polymer with a sufficiently high *ε*_yield_ and *ε*_crack_ must be selected, which may decrease upon blending with acceptor (*cf.* bulk-heterojunction blends; Section 4.3) or dopant molecules (*cf.* chemical doping; Section 5), but can also be enhanced through suitable additives such as polymeric binders.

Bulk devices where the organic semiconductor provides both the electrical as well as mechanical performance have been explored in the context of wearable electronics. For instance, conducting polymer tapes and fibers can function as electrical conductors, as actuators, as strain or electrochemical sensors (see Section 7.4), and they can be used as components in energy harvesting (*e.g.* thermoelectric generators; see Section 7.3) and storage devices (*e.g.* batteries, supercapacitors).^37^ Bulk materials must have a thickness of at least several μm so that they can handle the mechanical load without the support of a substrate. At first sight, bulk processing is straightforward because the electrical and mechanical properties of conjugated polymers tend to correlate (*cf.* Section 5.4). A wide range of conventional polymer processing methods can be readily utilized. Melt processing is often not feasible because of the prohibitively high melting temperatures of many conjugated polymers. Instead, solution processing methods such as wet spinning of fibers^37^ and 3D printing of gels^153^ are widely explored. However, processing of bulk materials as well as the operation of thick devices is limited by the rate of mass transport of auxiliary species such as solvent molecules, dopants, counterions, *etc.* For example, solution processing requires the removal of the processing solvent, which takes time if thick materials are to be created. The impact of the drying kinetics on nanostructure formation is well understood in case of thin films but is more difficult to control when bulk materials are prepared. As a result, there is a tendency for thin films to exhibit superior electrical properties compared with bulk materials.

### 7.2. Organic photovoltaics

**Fig. 18:**
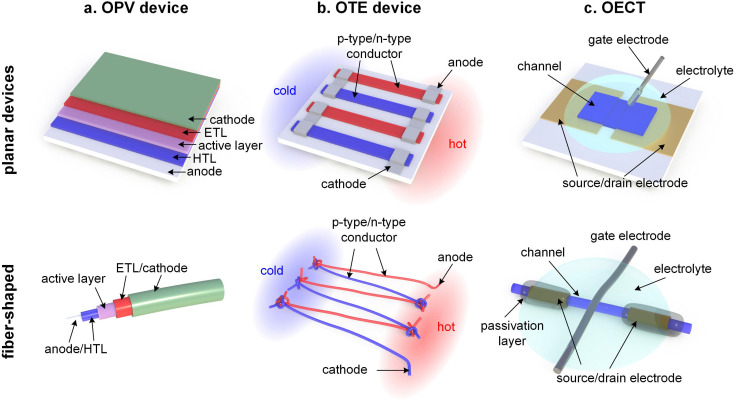
Planar thin-film (top row) and fiber-shaped bulk devices (bottom row), including (a) OPV devices that comprise an active layer sandwiched between an electron- and hole transport layer (ETL and HTL) as well as a cathode and anode; (b) OTE devices comprising alternating p- and n-type legs that are electrically connected in series but thermally in parallel; and (c) OECTs with an active layer that bridges the source and drain electrodes and is contact with a gate electrode *via* an electrolyte.

OPV devices, *i.e.* devices that generate an electrical potential when they absorb light, are selected as an example to illustrate the different material design strategies relevant for thin-film devices. OPV devices comprise an active layer composed of a mixture of one or several donor and acceptor materials, a so-called bulk-heterojunction blend, which is sandwiched between a cathode and an anode whose interfaces are typically modified by an electron- and a hole-transport layer, respectively (Fig. 18). One of the electrodes is transparent, letting light pass through that is absorbed by the active layer and converted into a photocurrent. The whole layer stack is supported by a substrate, which is often a rigid glass substrate if devices are manufactured for screening of new materials. Instead, flexible solar cells are usually manufactured on a planar plastic foil, *e.g.* made of poly(ethylene terephthalate) (PET),^154^ but can also be constructed with a non-planar geometry, *e.g.* using stainless steel wires,^155^ or by in-mold decoration through injection molding of thermoplastic polyurethane onto OPV modules on PET substrates.^156^

Deformation of devices on flexible substrates can cause mechanical degradation *via* adhesive failure between layers and cohesive failure of the active layer,^25^ and hence the selection of robust materials is critical for ensuring a stable performance.

During the last decade, the synthesis of new types of conjugated polymers has to a significant extent been fueled by the demand for new donor materials for organic photovoltaics. Most donor polymers have a high *T*_*g*_,^76^ which is thought to arrest (or at least slow down) phase separation of donor:acceptor bulk-heterojunction blends, resulting in a brittle material with a low *ε*_crack_. The addition of the acceptor – a fullerene derivative or a so-called non-fullerene acceptor (NFA) – tends to lead to further embrittlement because acceptors also tend to exhibit a high *T*_g_.^82,157^ Below the blend *T*_g_ (s), decohesion of the bulk-heterojunction active layer occurs *via* brittle failure, which can be mitigated by selecting a high-molecular weight polymer, as observed for P3HT:[6,6]-phenyl-C_61_-butyric acid methyl ester (PC_61_BM) devices.^158^

A number of approaches have been explored to improve the ductility of bulk-heterojunction blends including plasticizers,^25^ the addition of small-molecular additives that form an internal network^159^ and binder materials such as a polystyrene-*b*-poly(ethylene-*ran*-butylene)-*b*-polystyrene (SEBS) block copolymer (see Section 4).^160,161^ Moreover, the constitution of the conjugated polymer itself can be modified, *e.g.* through the introduction of a flexible spacer (Fig. 4), which increases the flexibility of the backbone and in the context of all-polymer OPV devices has yielded an *ε*_crack_ > 20%.^162^

### 7.3. Organic thermoelectrics

OTE devices, *i.e.* devices that generate an electrical potential when they experience a temperature gradient, are here selected as a case study to explore how chemically doped polymers are being utilized for wearable electronics. The most promising materials feature a high conductivity. Hence, the high doping regime is typically targeted (see eqn (11)), reaching a high charge-carrier density of up to 10^27^ m^−3^ (Fig. 1).^163^ Most fundamental research on organic thermoelectrics focuses on thin films on substrates since materials can be processed with the same printing and coating techniques that have been developed for, *e.g.*, OFETs and OPV devices. Thin films are characterized by applying an in-plane temperature gradient Δ*T*, while in practice devices are more likely to experience out-of-plane gradients. However, thick films or bulk materials are required for the construction of out-of-plane devices. In a thermoelectric device alternating legs of a p- and an n-type conductor are connected electrically in series and thermally in parallel (Fig. 18), and the device generates a thermovoltage when it experiences a Δ*T*. For devices based on conducting polymers the optimal leg thickness can be as high as 0.1 to 10 mm.^164^ Too thick devices have an unnecessarily high internal electrical resistance while a thin device only experiences a fraction of the available Δ*T* due to thermal contact resistance at the interfaces with the heat source and sink. Therefore, bulk materials are needed for the fabrication of thick thermoelectric devices, which must have an adequate mechanical robustness.^96^ Devices can be constructed with bulk materials that are solely based on conducting polymers, *e.g.* conducting polymer sheets or fibers, or they can be fabricated with coated sheets, fibers or yarns.^165^ Alternatively, the conducting polymer can be blended with an insulating polymer or reinforced with, *e.g.*, CNTs or graphene, which allows to adjust not only the electrical but also the rheological and mechanical properties (Section 4.3).^96^

To create bulk materials with conducting polymers, the processing method must be carefully selected. Co-processing of P3HT and F_4_TCNQ results in aggregation of the polymer in solution, and thus a brittle solid.^121^ Instead, millimeter-thick architectures of P3HT can be solid-state pressed, followed by sequential doping with F_4_TCNQ, which is however ineffective because of diffusion of the dopant is prohibitively low.^110^ Polymers such as p(g_4_2T-T) that show better compatibility with dopants such as F_4_TCNQ and H-TFSI can instead be shaped into bulk materials *via* co-processing from solution.^166^ One of the most promising p-type materials is PEDOT:PSS, which can be readily processed as an aqueous dispersion and has been utilized for the fabrication of free-standing films^167^ and fibers^7,168^ with a very promising thermoelectric performance.

Bulk materials such as silk and cellulose yarns coated with PEDOT:PSS^164,169^ have been used to fabricate thick textile devices by embroidering the conducting yarn into a wool fabric to create devices with a thickness of about 1 cm. Fused filament fabrication (FFF) 3D printing is another method to create thick out-of-plane devices. For example, a device with 100 leg pairs could be realized by first printing 1.6 mm thick, porous legs of a Nafion precursor on a textile substrate, which were then used as a template for the oxidative polymerization of PEDOT.^170^ Inkjet printing is being explored as a technique to combine solution processing with patterning of smaller leg pairs. For example devices with a leg thickness of 25 μm have been printed comprising PEDOT:PSS as the p-type material and a doped fullerene derivative as the n-type material.^171^ Finally, binder materials can be used to enhance the mechanical properties of thermoelectric materials, including semicrystalline polymers such as poly(ethylene oxide) PEO to increase the robustness of F_4_TCNQ doped P3HT^172^ and polyurethane to impart stretchability to PEDOT:PSS^173,174^ or p(g_4_2T-T).^175^

### 7.4. Organic electrochemical transistors

OECTs receive considerable interest as a type of device that allows to couple ionic and electronic current, which is of considerable interest for applications in bioelectronics, *i.e.* the integration of electronics with biological (living) systems.^4,68^ OECTs are here discussed as an example of a type of device that can either have a thin-film or bulk architecture and operates in the high doping regime, reaching *n* ≈ 10^27^ m^−3^ (see Fig. 1; conjugated polymer based devices operate at a gate voltage *V*_g_ < 1 V and some materials have a volumetric capacitance of up to C* ≈ 200 F cm^−3^).^68^ An OECT device comprises an organic mixed ionic–electronic conductor (OMIEC, *i.e.* a material with a suitably high ionic and electronic mobility), an electrolyte and, where applicable, a substrate. The device should match the mechanical properties of the tissue or biological material that it is in contact with, *e.g.* the elasticity of skin,^176^ the ultralow modulus of brain tissue^177^ or the stiffness of a plant xylem.^178^

The active layer of an OECT is composed of an OMIEC, which can be a conjugated polymer that is in contact with a gate electrode *via* an electrolyte, *e.g.* a salt dissolved in water or acetonitrile (Fig. 18). Application of a suitable electrical potential *V*_g_ at the gate electrode triggers a redox reaction in the polymer. Electronic charge is exchanged with the source or drain electrode and the generated charge is compensated through the exchange of ions with the electrolyte. The OMIEC can initially be a semiconductor or a conductor such as PEDOT:PSS, and the channel conductance is increased (accumulation mode) or decreased (depletion mode) during device operation. In both cases, ions accompanied by solvent molecules enter so that overall charge neutrality is maintained (see Section 6).

Thin-film devices can be deposited on a planar or curved substrate, including filaments and yarns, which makes OECTs ideally suited for textile-based logic circuits.^179^ Thin active layers (*d* < 100 nm) are preferred because the conductance of an OECT is altered upon changing *V*_g_. The device switching speed depends on the rate of ion exchange with the electrolyte, which is determined by both the ionic mobility and the thickness of the polymer layer. Hence, very thin layers maximize the switching speed for a given material, an important criterion for circuit design. The substrate can be rigid, flexible or elastic, and the polymer layer must be able to accommodate any imposed deformation. For instance, OECTs on a PDMS substrate based on p(g_3_2T-T) with an *M*_n_ = 68 kg mol^−1^ and *E* ≈ 50 MPa could be stretched to *ε* = 100% at least 5000 times without a significant change in device performance, which enabled positioning on skin for real-time recording of electrocardiogram (ECG) signals.^180^ Instead, PEDOT:PSS devices on a thermoplastic polyurethane substrate could only be stretched to *ε* = 50%, likely because of the higher stiffness of the active layer.^181^

OECTs can be deposited on a sacrificial substrate that can be used to position devices directly on skin or tissue, which takes over the role of the substrate.^176^ A significant part of the overall volume of an OECT is occupied by the electrolyte. Hence, to fabricate fully functional devices, it is important to use a mechanically robust electrolyte, which can be either a liquid (*e.g.* an aqueous electrolyte) or a solid electrolyte with suitable mechanical properties. The electrolyte can be used as the substrate, as demonstrated for devices on elastic gelatin hydrogel films with *E* < 1 MPa, on top of which meander-shaped PEDOT:PSS patterns were deposited.^182^ OECTs where a bulk material functions as both the channel and provides the mechanical integrity must have a thickness of at least a few micrometers. As a result, the rate of ion exchange and hence the switching speed is lower compared to thin-film devices. Ion ingression can be aided by maximizing the contact area between the channel material and the electrolyte through the use of a porous material. For instance, OECTs with a channel thickness of 1 mm could be created by dyeing the internal cell walls of balsa wood with PEDOT:PSS, which enabled ingression of the electrolyte and hence relatively fast switching on the order of a few seconds.^183^ Another approach is the use of non-planar architectures such as filaments. For example, oriented PEDOT:PSS microfibers with an *E* of up to 4 GPa have been used as the channel, yielding OECTs with a record device performance thanks to a very high *μ* of up to 13 cm^2^ V^−1^ s^−1^.^149^ Alternatively, a hydrophilic reinforcing agent such as CNF can be added to the conjugated polymer, which allows to modulate the mechanical properties and at the same time increases the ionic mobility.^79^

## 8. Outlook

The mechanical properties of only few classes of conjugated polymers such as polythiophenes have been studied in detail, meaning that most current knowledge is based on a handful of materials. Likewise, the mechanical properties of only few chemically doped polymers and hardly any electrochemically doped materials have been investigated. Further work is needed to elucidate the counteracting influence of changes in nanostructure (π-stacking) and backbone stiffness (persistence length, *T*_g_) *versus* the impact of swelling through the uptake of counterions and solvent molecules, especially in case of electrochemical doping but also changes in humidity. The development of methodologies that allow to monitor changes in mechanical properties *in situ* during (electro)chemical doping will be particularly insightful.

Many doped organic semiconductors are characterized by poor stability due to degradation reactions with, *e.g.*, water and oxygen. Moreover, unreacted dopants and counterions can diffuse and aggregate within thin-film layer stacks, leach out or sublime from devices and drift in an electric field, resulting in a change in not only the electrical but likely also mechanical properties of doped conjugated polymers. Further studies that explore the stability of doped materials are needed, as well as strategies that mitigate degradation reactions and hinder diffusion.

It can be anticipated that an in-depth understanding of the mechanical properties of (doped) conjugated polymers will enable the design of truly robust and/or elastic (semi)conducting materials, which promises to advance the fields of wearable electronics and bioelectronics. Moreover, the increased use of fatigue testing of conjugated polymers upon not only repeated electrical stress but also deformation is needed to realize materials that exhibit the long-term mechanical properties that are typical for engineering polymers. It can be anticipated that doping will be utilized as a tool to optimize the stiffness and ductility of conjugated polymers and may even allow the introduction of reversible behavior. One further opportunity is the more widespread use of the chemical toolbox developed in the context of thermoplastic elastomers and dynamic networks, which may facilitate the development of materials that not only feature attractive electrical and mechanical properties but can also be reused at the end of their lifetime.

## Conflicts of interest

There are no conflicts to declare.

## Supplementary Material
